# Circulating anti-Ro/Sjögren’s syndrome-related antigen A antibodies and risk of heart rate-corrected QT interval prolongation: a meta-analysis

**DOI:** 10.1093/ehjopen/oeag123

**Published:** 2026-08-14

**Authors:** Pietro Enea Lazzerini, Mark Markovich Melamud, Evgeny Alexandrovich Ermakov, Vadim Vyacheslavovich Konkov, Valentina Nikolaevna Buneva, Riccardo Accioli, Alessandra Cartocci, Anna Cantore, Viola Salvini, Decoroso Verrengia, Maurizio Acampa, Pier Leopoldo Capecchi, Mohamed Boutjdir

**Affiliations:** Department of Medical Sciences, Surgery and Neurosciences, University of Siena, Viale Bracci 14, Siena 53100, Italy; Division of Internal Medicine and Geriatrics, Electroimmunology Unit, University of Siena, Viale Bracci 14, Siena 53100, Italy; Laboratory of Repair Enzymes, Institute of Chemical Biology and Fundamental Medicine, Siberian Branch of the Russian Academy of Sciences, 8 Lavrentyev Avenue, Novosibirsk 630090, Russia; Laboratory of Repair Enzymes, Institute of Chemical Biology and Fundamental Medicine, Siberian Branch of the Russian Academy of Sciences, 8 Lavrentyev Avenue, Novosibirsk 630090, Russia; Department of Natural Sciences, Novosibirsk State University, 2 Pirogova Street, Novosibirsk 630090, Russia; Laboratory of Repair Enzymes, Institute of Chemical Biology and Fundamental Medicine, Siberian Branch of the Russian Academy of Sciences, 8 Lavrentyev Avenue, Novosibirsk 630090, Russia; Laboratory of Repair Enzymes, Institute of Chemical Biology and Fundamental Medicine, Siberian Branch of the Russian Academy of Sciences, 8 Lavrentyev Avenue, Novosibirsk 630090, Russia; Department of Natural Sciences, Novosibirsk State University, 2 Pirogova Street, Novosibirsk 630090, Russia; Department of Medical Sciences, Surgery and Neurosciences, University of Siena, Viale Bracci 14, Siena 53100, Italy; Division of Internal Medicine and Geriatrics, Electroimmunology Unit, University of Siena, Viale Bracci 14, Siena 53100, Italy; Division of Internal Medicine and Geriatrics, Electroimmunology Unit, University of Siena, Viale Bracci 14, Siena 53100, Italy; Division of Internal Medicine and Geriatrics, Electroimmunology Unit, University of Siena, Viale Bracci 14, Siena 53100, Italy; Department of Medical Sciences, Surgery and Neurosciences, University of Siena, Viale Bracci 14, Siena 53100, Italy; Division of Internal Medicine and Geriatrics, Electroimmunology Unit, University of Siena, Viale Bracci 14, Siena 53100, Italy; Department of Medical Sciences, Surgery and Neurosciences, University of Siena, Viale Bracci 14, Siena 53100, Italy; Division of Internal Medicine and Geriatrics, Electroimmunology Unit, University of Siena, Viale Bracci 14, Siena 53100, Italy; Department of Medical Sciences, Surgery and Neurosciences, University of Siena, Viale Bracci 14, Siena 53100, Italy; Division of Internal Medicine and Geriatrics, Electroimmunology Unit, University of Siena, Viale Bracci 14, Siena 53100, Italy; Department of Medical Sciences, Surgery and Neurosciences, University of Siena, Viale Bracci 14, Siena 53100, Italy; Division of Internal Medicine and Geriatrics, Electroimmunology Unit, University of Siena, Viale Bracci 14, Siena 53100, Italy; Department of Medicine, Cell Biology and Pharmacology, Cardiovascular Research Program, VA New York Harbor Healthcare System, SUNY Downstate Health Sciences University, 445 Lenox Rd, Brooklyn, New York, NY 11203, USA; Department of Medicine, New York University Grossman School of Medicine, 550 First Avenue, New York, NY 10016, USA

**Keywords:** Anti-Ro/SSA antibodies, QTc prolongation, QTc values, Connective tissue diseases, Anti-Ro/SSA-52kD antibodies

## Abstract

**Aims:**

Accumulating evidence indicates that anti-Ro/Sjögren’s syndrome-related antigen A (SSA) antibodies, specifically the anti-Ro/SSA-52kD subtype, can promote heart rate-corrected QT interval (QTc) prolongation by inhibiting the hERG-K^+^ channel. However, the numerous clinical studies published over the past two decades yielded conflicting results. This meta-analysis aims at pooling the available data to provide clarity on this clinically relevant issue.

**Methods and results:**

A systematic review was performed for clinical studies comparing QTc in anti-Ro/SSA-positive vs. -negative subjects. Standardized mean differences (SMDs) and odds ratios (ORs) were compared using random effects models. The risk of bias was assessed using RoBANS2, Egger's test and contour-enhanced funnel plots. Twenty-five studies were included, pooling 2122 anti-Ro/SSA-positive and 8865 anti-Ro/SSA-negative subjects. Overall, anti-Ro/SSA-positivity is associated with a longer QTc [SMD = 0.36 (0.18–0.53), *P* < 0.001], and a more than two times higher risk of QTc prolongation [OR = 2.24 (1.54–3.27), *P* < 0.001]. Several sub-analyses supported and refined these findings. Specifically, in anti-Ro/SSA-positive patients with unselected connective tissue disease, QTc absolute values are higher [SMD = 0.56 (0.20–0.93), *P* = 0.0026] and QTc prolongation occurs more frequently [OR = 6.99 (2.35–20.75), *P* < 0.001] than in negative patients; furthermore, QTc prolongation is also more common in systemic lupus erythematosus patients with circulating anti-Ro/SSA-antibodies than in those without [OR = 1.86 (1.19–2.90), *P* = 0.006]. Finally, in anti-Ro/SSA-52kD-positive subjects, the QTc is longer compared to the negative ones [SMD = 0.43 (0.15–0.71), *P* = 0.002].

**Conclusion:**

This meta-analysis supports the conclusion that circulating anti-Ro/SSA-antibodies *per se* represents a significant risk factor for QTc prolongation. However, the presence of several conflicting studies suggests that a more accurate investigation of specific factors, such as the impact of individual antibody subtype levels (anti-52kD/anti-60kD), will be critical to fully clarify the topic and its implications in clinical practice.

## Structured Graphical Abstract

**Structured Graphical Abstract:**
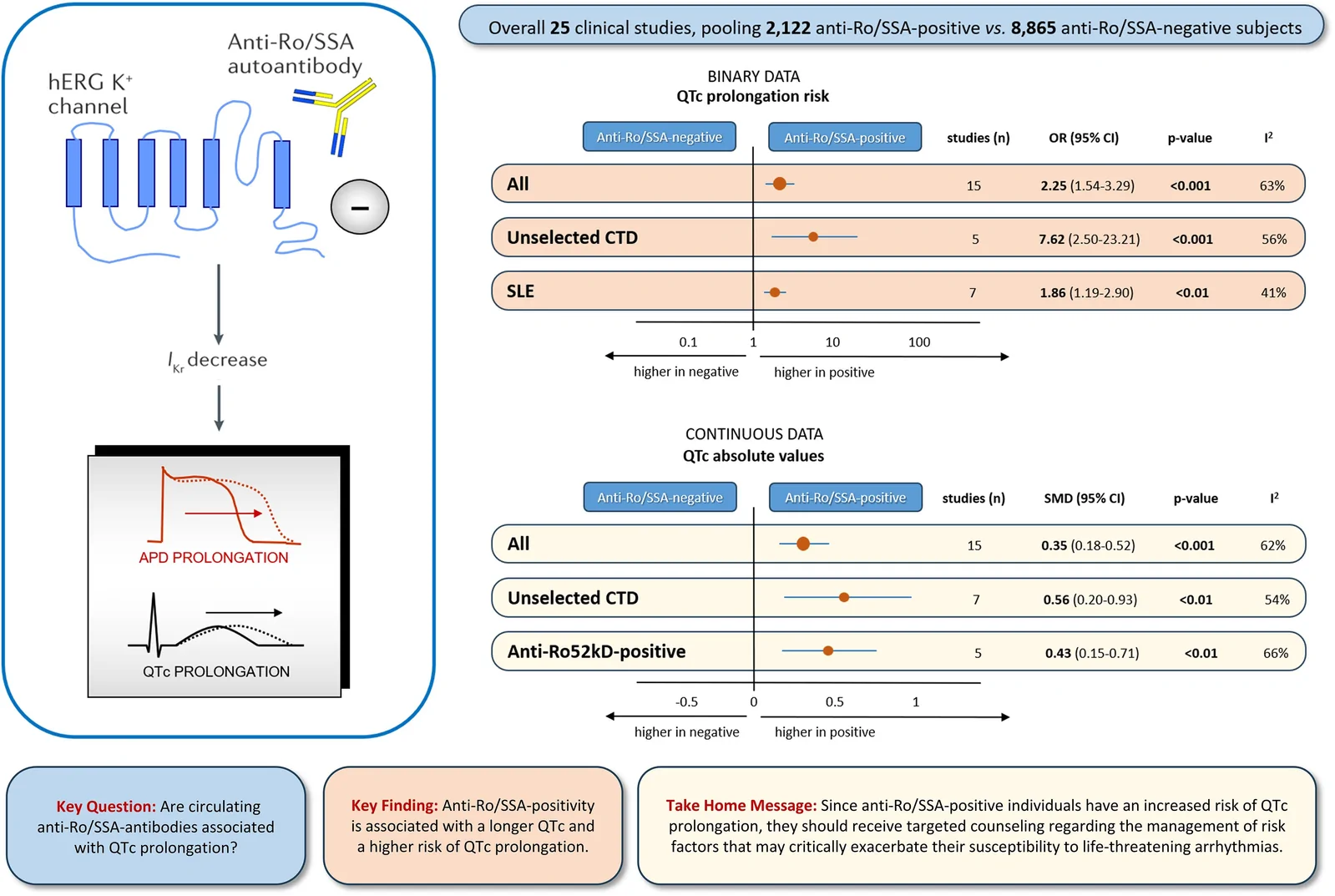

## Introduction

Long QT syndrome (LQTS) is a cardiac electric disorder characterized by an abnormal prolongation of the heart rate-corrected QT interval (QTc), increasing susceptibility to malignant ventricular arrhythmias, specifically torsades de pointes (TdP) ventricular tachycardia.^1^ While TdP risk progressively increases when the QTc exceeds 440 ms, becoming high at values >500 ms and very high at >600 ms, in the last decades, many different cut-offs were recommended by scientific societies to define QTc prolongation as abnormal. They include: (i) >440 ms regardless of sex, representing the ‘classic’ cut-off for QTc prolongation; (ii) >450 ms in males or >460 in females; and (iii) >470 ms in males or >480 in females, representing approximate 99th percentile QTc values for otherwise healthy post-pubertal individuals.^1,2^

LQTS has many potential causes, either inherited, owing to variants in genes encoding potassium, sodium, or calcium channels (*genetic cardiac channelopathies*), or acquired.^1^ Among acquired forms, the most common causes are medications that block the human *ether-à-go-go*-related gene (hERG) potassium channel, which conducts the rapid component of the delayed rectifier potassium current I_Kr_, and electrolyte imbalances.^1^ More recently, substantial evidence points to autoimmunity as a novel and epidemiologically relevant cause of acquired LQTS, due to circulating anti-ion channel autoantibodies interfering with cardiac electrophysiology (*autoimmune cardiac channelopathies*).^3,4^

Anti-Ro/Sjögren’s syndrome-related antigen A (SSA) antibodies, including anti-Ro/SSA-52kD and anti-Ro/SSA-60kD subtypes, result from an autoimmune response targeting the Ro-ribonucleoprotein, constituted by two subunits of 52 and 60 kD, respectively.^5^ While anti-Ro/SSA antibodies are frequently detected in patients with autoimmune diseases, particularly connective tissue diseases (CTDs) such as systemic lupus erythematosus (SLE) and Sjogren syndrome (SS),^5^ they can be also found in individuals from general population (0.5–8% depending on age).^6,7^ Accumulating clinical and experimental studies provided evidence that anti-Ro/SSA antibodies, specifically anti-Ro/SSA-52kD subtype, can cause LQTS by cross-reacting with and inhibiting hERG, due to a sequence homology between Ro52kD antigen and the extracellular loop of the channel.^8–10^ This leads to I_Kr_, inhibition, resulting in ventricular repolarization slowing and QTc prolongation.^8^ However, the published clinical studies to date do not provide a definite answer to the question: do anti-Ro/SSA antibodies significantly affect the QTc values? In the early stages of this research topic, some studies confirmed the effect of anti-Ro/SSA antibodies on QTc,^11,12^ but some others did not.^13,14^ This trend continues to the present day.

The above question can be approached from two perspectives, i.e. by evaluating whether: (i) there is an association between the presence of anti-Ro/SSA antibodies and absolute QTc values, and (ii) there is an association between the presence of anti-Ro/SSA antibodies and QTc prolongation occurrence. While the first perspective is straightforward, the second one introduces uncertainty, as different studies used different cut-offs for QTc prolongation, including 430 ms,^15^ 440 ms,^11,12^ 460 ms,^16^ 470 ms in males/480 in females,^7^ and 500 ms.^17^

The complexity of this issue is further compounded by the heterogeneity of anti-Ro/SSA antibodies, which include two subtypes, only one of which appears to be arrhythmogenic. In fact, current evidence indicates that only the anti-Ro/SSA-52kD subtype can promote QTc prolongation due to its specific cross-reactivity with hERG,^8,9,16,18^ a blocking effect whose magnitude directly depends on the patient’s circulating levels.^16,19^ Nevertheless, most existing studies only consider overall anti-Ro/SSA positivity, irrespective of subtype or titre, a limitation that likely contributes to the contradictory and ambiguous results reported.

These observations indicate that the impact of anti-Ro/SSA antibodies on QTc is modulated by multiple factors, thereby complicating the interpretation of this association. Key uncertainties remain, including whether anti-Ro/SSA antibodies affect the absolute QTc duration (ms), the prevalence of QTc prolongation (%), or both. Additional questions concern the most appropriate threshold for defining QTc prolongation and whether antibody subtypes particularly anti-Ro/SSA-52kD and anti-Ro/SSA-60kD exert differential effects on ventricular repolarization. Despite the substantial number of studies addressing this topic, no meta-analysis has systematically evaluated these issues. The present study was therefore undertaken to address this important gap in the literature.

Another unresolved issue is whether maternal anti-Ro/SSA antibodies impact QTc in infants. Although fewer studies have examined this question than in adults, it remains clinically important but again the available evidence is contradictory.^20–24^ This is another unmet need that the present meta-analysis seeks to also address.

Thus, the objective of this study was to perform a systematic review and meta-analysis to evaluate whether the presence of circulating anti-Ro/SSA antibodies is associated with a longer QTc and/or higher risk of QTc prolongation compared with their absence.

## Methods

### Study search

This meta-analysis was performed following the Preferred Reporting Items for Systematic Reviews and Meta-Analyses (PRISMA 2020) guidelines. The search for eligible articles was carried out in PubMed/MEDLINE, Google Scholar, and Scopus databases. The time frame for the literature search was from 1958 (the first mention of anti-Ro/SSA antibodies) to May 2025. Search queries for databases are presented in Supplementary material online, Appendix pp. 3–4. A reference list of selected articles was screened to identify additional studies. Only peer-reviewed published articles were considered. Conference abstracts and editorials were not included in the study. Three investigators (P.E.L., M.M.M., and E.A.E.) independently reviewed the results of each query and then assessed the relevance of all retrieved articles. The final reference list was compiled after reaching consensus with all co-authors.

### Eligibility criteria

The following inclusion criteria were used in this meta-analysis: (i) the study (or part of it), regardless of design, should be devoted to measure QTc values or detect presence/absence of QTc prolongation in the presence/absence of anti-Ro/SSA antibodies; and (ii) the study must be indexed in at least one of the international databases. The meta-analysis included studies published in English, or other languages with English translations. The studies were then divided into several subgroups depending on: (i) the age of the subjects involved (adults/infants), (ii) the modality of data presentation (binary/continuous), (iii) the patients’ diagnosis (unselected CTD/SLE), and (iv) the antibody subtype investigated (anti-Ro/SSA-52kD/-60kD), in all cases provided that at least three studies were available. Moreover, again if at least three studies were available, additional sub-analyses were performed focusing on specific QTc prolongation cut-offs used.

### Data extraction and statistical analysis

Three independent investigators (P.E.L., M.M.M., and E.A.E.) extracted data from the retrieved articles. Once the list of studies was approved for inclusion, their quality was checked using the STROBE (STrengthening the Reporting of OBservational studies in Epidemiology) authors’ guide. More information is available at https://www.strobe-statement.org/ (STROBE Checklist: cross-sectional studies). The results were analysed using MetaAnalysesOnline. Standardized mean differences (SMDs) and odds ratios (ORs) were compared using a random effects model with inverse variance method. The degree of heterogeneity was assessed using the *I*^2^ statistics test. *I*^2^ values of 0–25%, 26–50%, 51–75%, and 75–100% were classified as indicating no, low, moderate, and substantial heterogeneity, respectively. Each study included in the meta-analysis was then assessed for level of bias. The assessment was performed using the Revised Risk of Bias Assessment Tool for Nonrandomized Studies (RoBANS2).^25^ For details of the bias algorithm, see Seo *et al*.^25^ The level of bias was assessed using Egger’s test and contour-enhanced funnel plots (CEFP). CEFP was constructed via R code using meta package and deposited in GitHub (https://bit.ly/46ypInq). For more information about the interpretation of CEFP, see Peters *et al*.^26^ Meta-regression analysis was performed using Numpy, Pandas, and Statsmodels Python libraries. The code used is deposited on GitHub (https://bit.ly/4rKqO80).

## Results

### Reports selection

The PRISMA flowchart diagram describing the conducted report selection is presented in *Figure 1*. A primary search of the databases yielded a total of 1136 reports (238, 396, and 502 from Google Scholar, PubMed, and Scopus, respectively), and 190 reports were removed as duplicates. Of the 944 titles and abstracts reviewed, 919 reports were removed because 55 were reviews, 30 were not written in English (nor translated), and 834 were out of scope. Twenty-seven reports were further retrieved for thorough evaluation, and two of them were excluded because the comparison groups were intentionally composed only of patients with QTc prolongation or TdP, a design that would have introduced substantial selection bias into the present meta-analysis.^8,9^ Ultimately, 25 reports were selected, overall including 2122 anti-Ro/SSA-positive and 8865 anti-Ro/SSA-negative individuals.

**Figure 1:**
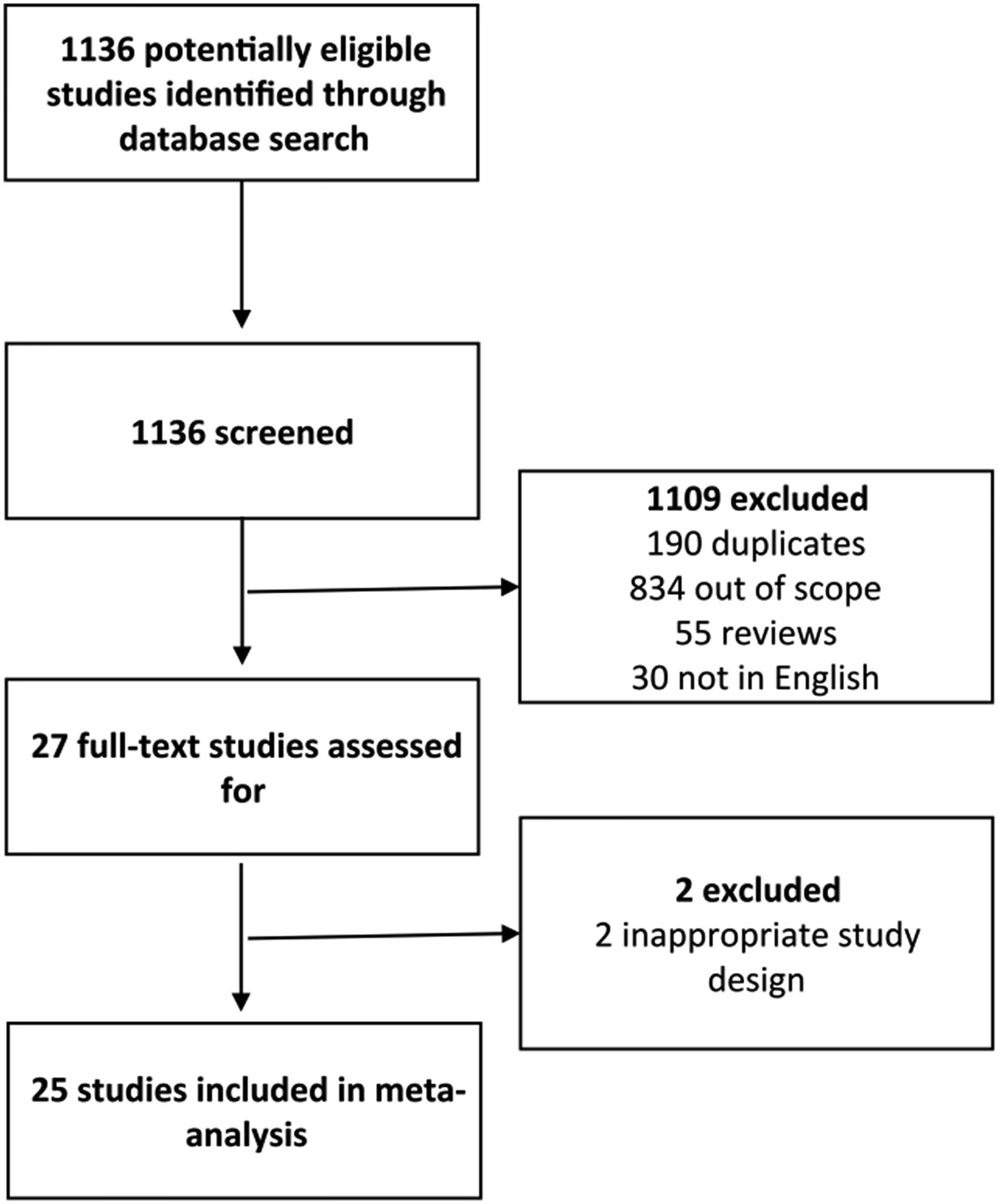
PRISMA flowchart diagram of report screening and selection process.

### Reports characteristics

The list of studies used for meta-analysis is presented in *Table 1*. As all 25 studies were cross-sectional by design, they underwent and passed the quality control checks using the Joanna Briggs Institute (JBI) Critical Appraisal Checklist for analytical cross-sectional studies (last amended in 2017).^37^ Detailed information on the results of the quality assurance review is available in Supplementary material online, Appendix pp. 6–24.

**Table 1:** Characteristics of the clinical studies included in the meta-analysis

Study	Year	Diagnosis	Data type	QTc prolongation cut-offs, ms	Age population	Anti-Ro/SSA +/− subjects, *n*	Anti-Ro/SSA subtype testing, anti-52kD/anti-60kD	Anti-Ro/SSA +/− female, %	Anti-Ro/SSA +/− under antimalarial therapy, %
Gordon *et al*. ^27^	2001	SLE	Continuous	NA	Adults	49/62	NA/NA	100/100	NA/NA
Lazzerini *et al*.^11^	2004	Unselected CTDs	Binary/continuous	440	Adults	31/26 31/26	NA/NA	97/85	35.5/19.2
Costedoat-Chalumeau *et al*.^13^	2005	Unselected CTDs	Binary/continuous	440	Adults	31/55 32/57	NA/NA	91/95	100/100
Cardoso *et al*.^28^	2005	SLE	Continuous	NA	Adults	14/35	NA/NA	100/100	NA/NA
Lazzerini *et al*.^29^	2007	Unselected CTDs	Binary/continuous	440	Adults	26/20 26/20	NA/NA	92/90	15.4/40
Bourré-Tessier *et al*.^12^	2011	SLE	Binary	440	Adults	114/164	NA/NA	NA/NA	NA/NA
Lazzerini *et al*.^16^	2011	Unselected CTDs	Binary/continuous	460	Adults	25/24 25/24	Yes/Yes	84/88	36/41.7
Nomura *et al*.^15^	2014	SLE	Binary	430	Adults	43/47	NA/NA	NA/NA	NA/NA
Teixeira *et al*.^17^	2014	SLE	Binary	500	Adults	113/204	NA/NA	NA/NA	NA/NA
Massie *et al*.^14^	2014	SSc	Binary	440	Adults	148/541	Yes|Yes	NA/NA	NA/NA
Pisoni *et al*.^30^	2015	Unselected CTDs	Binary/continuous	440	Adults	55/18 55/18	NA/NA	93/82	56.4/70.6
Bourré-Tessier *et al*.^31^	2015	SLE	Binary/continuous	440; 460	Adults	283/314 283/314	Yes|Yes	NA/NA	NA/NA
Tufan *et al*.^18^	2017	Unselected CTDs	Continuous	NA	Adults	15/39	Yes/NA	100/95	60/71.8
Mostafavi *et al*.^32^	2020	SLE	Binary	440	Adults	55/95	NA/NA	NA/NA	NA/NA
Azharudeen *et al*.^33^	2020	SLE	Binary/continuous	440	Adults	63/77 63/77	NA/NA	NA/NA	NA/NA
Villuendas *et al*.^34^	2021	SLE	Binary/continuous	450 (460)	Adults	46/99 46/99	NA/NA	91/92	NA/NA
Lazzerini *et al*.^7^	2021	General population	Binary/continuous	470 (480); 490; 500	Adults	612/6727 612/6727	NA/NA	26/20	48.9/6.2
Gamazo-Herrero *et al*.^19^	2024	Unselected CTDs	Binary/continuous	440	Adults	101/66	Yes/NA	88/77	47.5/34.8
Wang *et al*.^35^	2024	DM	Continuous	NA	Adults	59/222	Yes|No	NA/NA	NA/NA
Ösken *et al*.^36^	2025	SS	Continuous	NA	Adults	32/25	NA/NA	96/94	NA/NA
Cimaz *et al*.^20^	2000	Children from CTD mothers	Continuous	NA	Infants	21/7	NA/NA	NA/NA	NA/NA
Gordon *et al*.^22^	2001	Children from CTD mothers	Continuous	NA	Infants	38/7	NA/NA	NA/NA	NA/NA
Costedoat-Chalumeau *et al*.^23^	2004	Children from CTD mothers	Continuous	NA	Infants	58/27	NA/NA	NA/NA	NA/NA
Gerosa *et al*.^24^	2007	Children from CTD mothers	Continuous	NA	Infants	46/17	NA/NA	NA/NA	NA/NA
Motta *et al*.^21^	2007	Children from CTD mothers	Continuous	NA	Infants	43/44	NA/NA	NA/NA	NA/NA

The ‘anti-52kD/anti-60kD’ column indicates whether separate subsets of anti-Ro/SSA-52kD and anti-Ro/SSA-60kD autoantibodies are being studied. The cut-off for women is indicated in parentheses if the study had different cut-offs for men and women. Semicolons separate different cut-offs within the same study.

CTDs, connective tissue diseases; DM, dermatomyositis; NA, not applicable or not available (in the columns for sex and drug therapy, NA also denotes those studies where such data are available but cannot be applied to the groups based on the presence or not of anti-Ro/SSA antibodies in the blood); SLE, systemic lupus erythematosus; SSc, systemic sclerosis; SS, Sjögren syndrome.

Once the final list of studies was approved, their quality was checked using the STROBE authors’ guide.^38^ The results are summarized in Supplementary material online, Appendix p. 25. Each study included in the meta-analysis was then assessed for level of bias using RoBANS2. The results are depicted in Supplementary material online, Appendix p. 26.

The study results were presented in two different ways: continuous and binary. Continuous study results answer the question of how much longer the QTc is in anti-Ro/SSA-positive than in anti-Ro/SSA-negative individuals. Binary study results answer the question of how much more often anti-Ro/SSA-positive individuals develop QTc prolongation compared to anti-Ro/SSA-negative individuals. A total of 15 studies were found that presented results as binary data,^7,11–17,19,29–34^ while 20 presented results as continuous data.^7,11,13,16,18–24,27–31,33–36^ Since studies presenting binary data used different cut-offs for defining QTc prolongation, detailed information on cut-off values for individual studies is provided in *Table 1*.

Anti-Ro/SSA antibodies are most often detected in patients with CTDs, and their effect on the QTc may not be the same in different CTD types. Therefore, we divided the studies according to the specific CTD associated (*Table 1*).

Most studies included in this meta-analysis examined total anti-Ro/SSA antibodies. However, some of them focused on specific subtypes. Six publications reported data on anti-Ro/SSA-52kD antibodies,^14,16,18,19,31,35^ while three on anti-Ro/SSA-60kD antibodies^14,16,31^ (*Table 1*).

A total of five studies investigating the effect of maternal anti-Ro/SSA antibodies on the QTc were found, all of which presented results as continuous data^20–24^ (*Table 1*).

Patient sex also may affect QTc values, due to well-known hormonal influences on ventricular repolarization.^1,39^ This aspect may be relevant considering that anti-Ro/SSA positivity is more common in females.^5,6^ Sex-specific data suitable for meta-analysis were identified in 12 studies (*Table 1*).

Another important factor that can influence QTc values is concomitant drug treatment.^1^ Therapy data suitable for meta-analysis were identified in eight studies, which reported various medications potentially prolonging the QTc. However, only antimalarial drugs, indicated as ‘antimalarials’, ‘chloroquine’, or ‘hydroxychloroquine’, were reported across all eight studies, as they represent the class of QT-prolonging drugs most frequently used in CTD patients^40^ (*Table 1*).

Based on the above premises, the impact of sex proportion and antimalarial medication use on QTc values was assessed by performing two specific meta-regression analyses.

### Continuous data outcomes

All publications with a continuous data outcome were analysed, i.e. studies addressing the question: how much QTc values differ between subjects with vs. without anti-Ro/SSA antibodies? The analyses were performed for the following subgroups (*Figure 2A–D*): (i) all available studies with a continuous data outcome; (ii) studies involving patients with unselected CTDs; (iii) studies involving SLE patients; (iv) studies involving patients tested for the anti-Ro/SSA-52kD antibody subtype; and (v) studies involving infants from anti-Ro/SSA-positive mothers.

**Figure 2:**
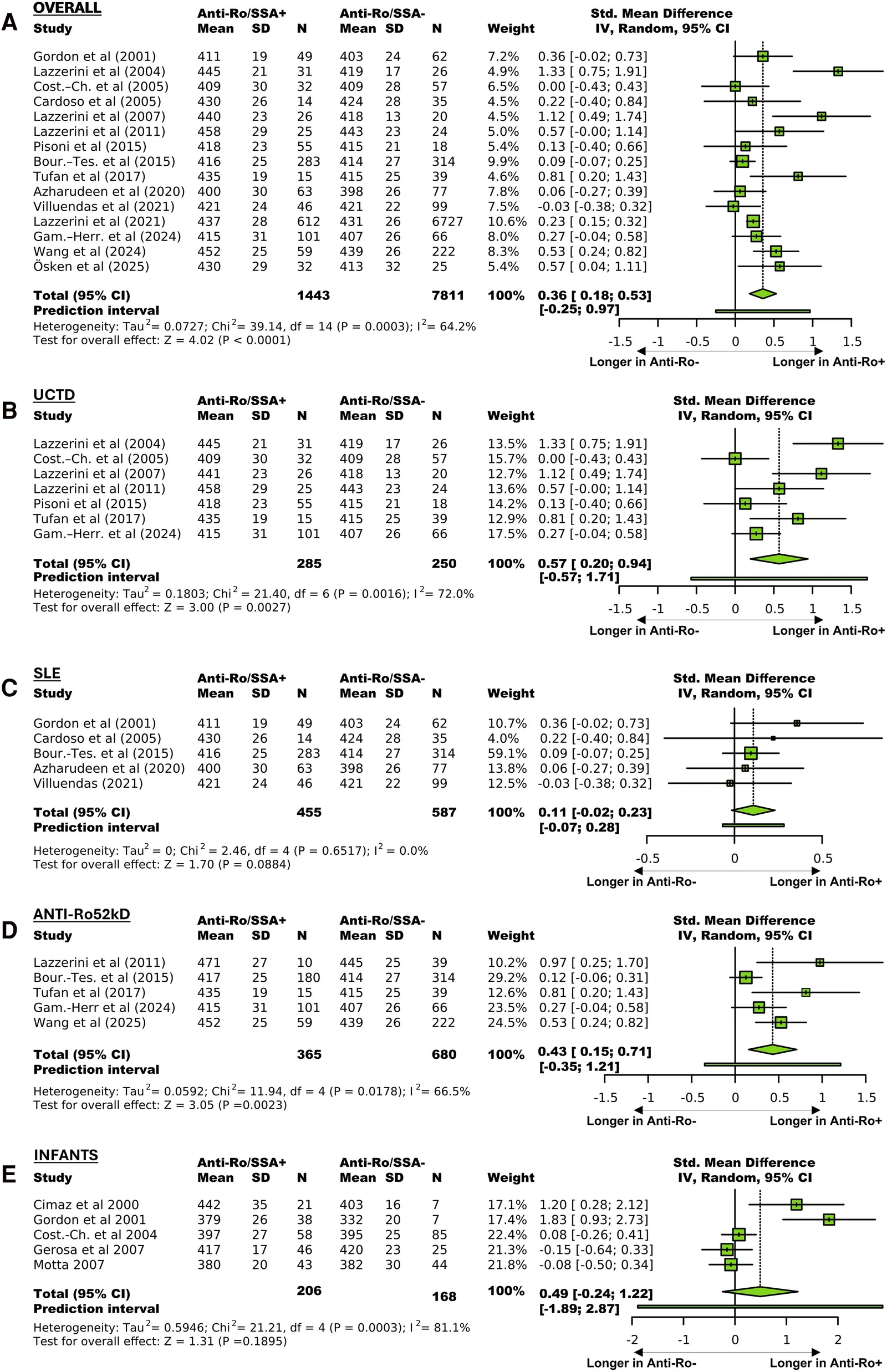
Forest plot of the standardized mean difference of QTc values in anti-Ro/SSA-positive vs. anti-Ro/SSA-negative individuals. *(A)* Analysis for all the available studies overall; *(B)* analysis for studies involving patients with unselected connective tissue diseases; *(C)* analysis for studies involving patients with systemic lupus erythematosus; *(D)* analysis for studies involving patients tested for anti-Ro/SSA-52kD antibody subtype; *(E)* analysis for studies involving infants from anti-Ro/SSA-positive mothers. SD: standard deviation; *N*: number of patients in a group; CI: confidence interval; IV: inverse variance; Random: random effects model; SLE: systemic lupus erythematosus; UCTD: unselected connective tissue diseases.

The presence of circulating anti-Ro/SSA antibodies was found to be associated with a statistically significant increase of QTc values when all available studies were considered overall (SMD = 0.36 [0.18–0.53]; *P* < 0.001) (*Figure 2A*), as well as when studies involving patients with unselected CTDs (SMD = 0.57 [0.20–0.94]; *P* = 0.002) (*Figure 2B*), or involving patients tested for the anti-Ro/SSA-52kD antibody subtype (SMD = 0.43 [0.15–0.71]; *P* = 0.0023) (*Figure 2D*) were specifically considered. Conversely, no statistically significant association was found in studies involving SLE patients (SMD = 0.11 [−0.02 to 0.23]) (*Figure 2C*) and in studies involving infants from anti-Ro/SSA-positive mothers (SMD = 0.49 [−0.24 to 1.22]) (*Figure 2E*).

No publication bias was detected when all available studies were considered together [Egger’s test, *P* = 0.101; CEFP (see Supplementary material online, Appendix p. 27)], as well as when studies involving patients with unselected CTDs [Egger’s test, *P* = 0.09; CEFP (see Supplementary material online, Appendix p. 28)], patients with SLE [Egger’s test, *P* = 0.61; CEFP (see Supplementary material online, Appendix p. 29)], or infants from anti-Ro/SSA-positive mothers [Egger’s test, *P* = 0.08; CEFP (see Supplementary material online, Appendix p. 32)] were selectively considered. However, Egger’s test revealed evidence of publication bias for studies involving patients tested for the anti-Ro/SSA-52kD antibody subtype (*P* = 0.04), a finding confirmed by the CEFP (see Supplementary material online, Appendix p. 30).

### Binary data outcomes

All publications with a binary data outcome were then analysed, i.e. studies addressing the question: how much the frequency of QTc prolongation differ between patients with and without anti-Ro antibodies? The analyses were performed for the following subgroups (*Figure 3A–G*): (i) all available studies with a binary data outcome regardless of the cut-off used; (ii) studies using a 440-ms cut-off; (iii) studies using a 460-ms or greater cut-off; (iv) studies involving patients with unselected CTDs; (v) studies involving SLE patients; (vi) studies involving patients tested for the anti-Ro/SSA-52kD antibody subtype; and (vii) studies involving patients tested for the anti-Ro/SSA-60kD antibody subtype.

**Figure 3:**
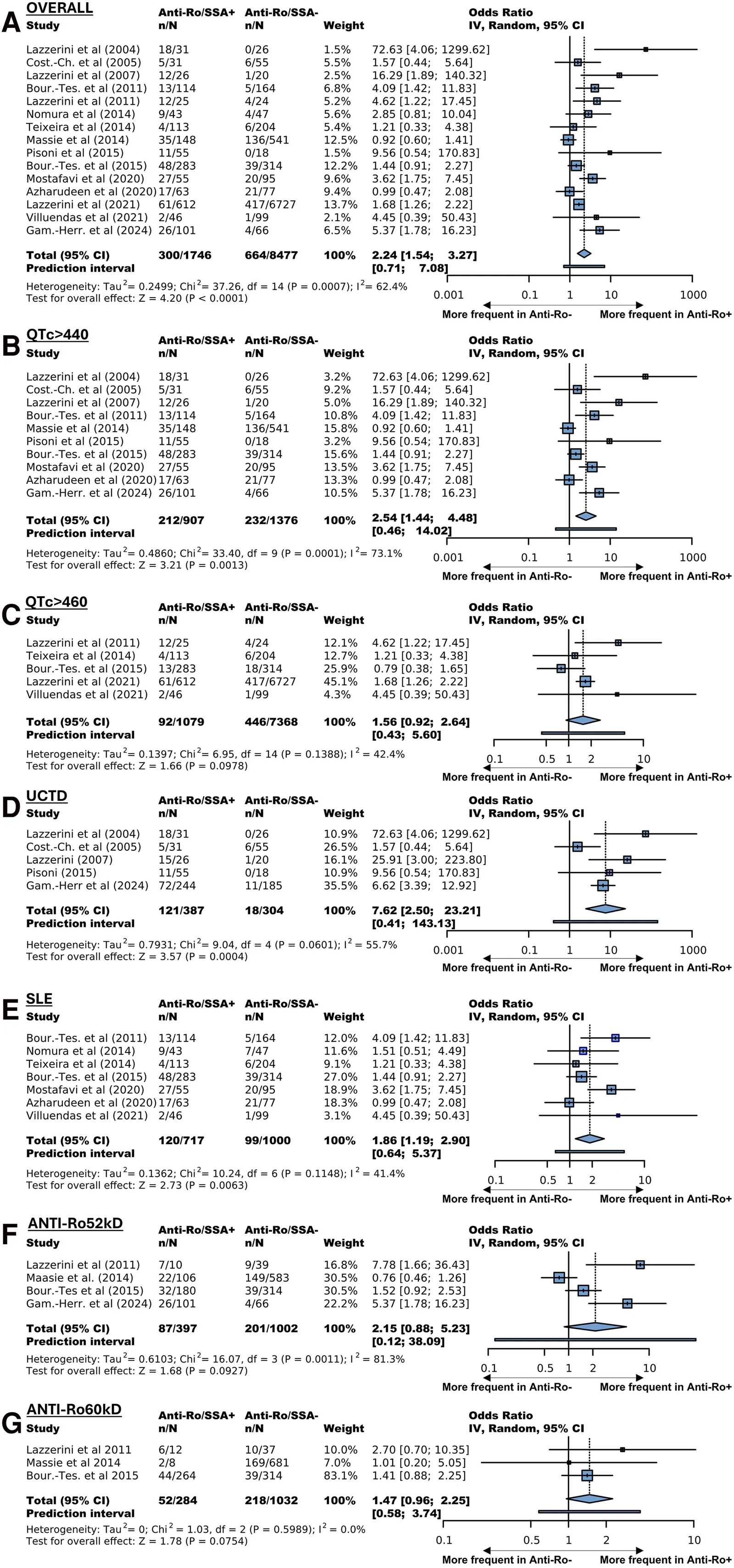
Forest plots of the odds ratio of QTc prolongation in anti-Ro/SSA-positive vs. anti-Ro/SSA-negative individuals. *(A)* Analysis for all the available studies overall, regardless of the specific cut-off used; *(B)* analysis for studies using a 440-ms cut-off; *(C)* analysis for studies using a 460-ms cut-off or greater; *(D)* analysis for studies involving patients with unselected connective tissue diseases; *(E)* analysis for studies involving patients with systemic lupus erythematosus; *(F)* analysis for studies involving patients tested for anti-Ro/SSA-52kD antibody subtype; *(G)* analysis for studies involving patients tested for anti-Ro/SSA-60kD antibody subtype. SD: standard deviation; *n*: cases with QTc prolongation; *N*: group size; CI: confidence interval; IV: inverse variance; Random: random effects model; SLE: systemic lupus erythematosus; UCTD: unselected connective tissue diseases.

A statistically significant association between the presence of circulating anti-Ro/SSA antibodies and a higher risk of QTc interval prolongation was found when all available studies were considered overall (OR = 2.24 [1.54–3.27]; *P* < 0.001) (*Figure 3A*), as well as when studies using a cut-off of 440 ms (OR = 2.54 [1.44–4.48]; *P* = 0.0013) (*Figure 3B*), involving patients with unselected CTDs (OR = 7.62 [2.50–23.21]; *P* < 0.001) (*Figure 3D*), or patients with SLE (OR = 1.86 [1.19–2.90]; *P* = 0.0063) were selectively considered. On the contrary, no statistically significant association was found in studies using with a cut-off of 460 ms or greater (OR = 1.56 [0.92–2.64]) (*Figure 3C*), as well as in those involving patients tested for the anti-Ro/SSA-52kD antibody subtype (OR = 2.15 [0.88–5.23]) (*Figure 3F*) or patients tested for the anti-Ro/SSA-60kD antibody subtype (OR = 1.47 [0.96–2.25]) (*Figure 3G*).

No publication bias was detected when studies using a cut-off of 460 ms or greater [Egger’s test, *P* = 0.921; CEFP (see Supplementary material online, Appendix p. 27)], as well as when studies involving patients with SLE [Egger’s test, *P* = 0.52; CEFP (see Supplementary material online, Appendix p. 29)], patients tested for the anti-Ro/SSA-52kD antibody subtype [Egger’s test, *P* = 0.1; CEFP (see Supplementary material online, Appendix p. 30)], or patients tested for the anti-Ro/SSA-60kD antibody subtype [Egger’s test, *P* = 0.99; CEFP (see Supplementary material online, Appendix p. 31)] were specifically considered. In the case of studies involving patients with unselected CTDs, Egger’s test revealed no evidence of publication bias (*P* = 0.1), although CEFP indicates potential publication bias (see Supplementary material online, Appendix p.28). There were clearer indications of possible publication bias when all available studies with a binary data outcome were considered overall (Egger’s test, *P* = 0.019), and in those selectively involving patients with a 440-ms cut-off (Egger’s test, *P* = 0.01), findings in both cases confirmed by the CEFP (see Supplementary material online, Appendix p. 25).

### Meta-regression analysis

A meta-regression analysis was conducted to identify the potential influence of certain important covariates on the analysis results. The meta-regression analysis was conducted for 13 studies with binary data outcomes^11–13,15–17,19,29–34^ and for 12 studies with continuous data outcomes,^13,16,18,19,23,27–31,33,34^ in all cases including only patients with unselected CTDs or SLE. The meta-regression analysis revealed that neither SMD (for continuous data) nor OR (for binary data) showed significant association with publication year or study population size. However, a significant association was found between OR and patient diagnosis categories. Studies with unselected CTDs demonstrated significantly higher OR values compared to SLE studies (*P* = 0.028). No such association was observed for SMD (*Table 2*). Consistent data were obtained when one single predictor (i.e. diagnosis) was investigated for binary data outcomes (adjusted *R*^2^ = 0.325, *t* = −2.602, *P* = 0.025). Of note, low heterogeneity was observed (31%) in these 13 studies.

**Table 2:** Meta-regression analysis results for both binary and continuous outcome data of studies including patients with unselected CTD or SLE

Variable	Coefficient	SE	*t*	*P*-value	Adjusted *R*^2^
Binary data outcomes (*n* = 13)					
Year	0.0812	0.050	1.628	0.138	0.365
Population	0.0002	0.002	0.108	0.916	
Diagnosis	−1.8190	0.698	−2.608	**0**.**028**	
Continuous data outcomes (*n* = 12)					
Year	−0.0046	0.014	−0.320	0.757	−0.288
Population	−0.0003	0.001	−0.475	0.647	
Diagnosis	−0.0207	0.225	−0.092	0.929	

The dependent variables were standardized mean difference for continuous outcomes and odds ratio for binary outcomes. Meta-regression was performed using weighted least squares estimation. *t* (*t*-statistic) is calculated as the regression coefficient divided by its SE. Adjusted *R*^2^ indicates the proportion of variance in the effect sizes across studies that is explained by the meta-regression model. *n* indicates number of studies included. Statistically significant *P-*values are reported in bold.

CTD, connective tissue disease; SLE, systemic lupus erythematosus; SE, standard error.

In addition, two separate meta-regression analysis was performed to assess the influence of sex proportion and antimalarial medication use on QTc values, and both ones revealed no statistically significant effect of these covariates (*Table 3*).

**Table 3:** Meta-regression analysis results for continuous outcome data of studies providing information regarding sex distribution or concomitant treatment with antimalarial drugs

Variable	Coefficient	SE	*t*	*P*-value	Adjusted *R*^2^
Sex (*n* = 12)					
Female sex	0.1006	0.4806	0.2093	0.8384	−0.0952
Antimalarial drug treatment (*n* = 8)					
Antimalarial drug use	−0.5104	0.5787	−0.8819	0.4118	−0.0328

The dependent variables were standardized mean difference. Meta-regression was performed using weighted least squares estimation. *t* (*t*-statistic) is calculated as the regression coefficient divided by its SE. Adjusted *R*^2^ indicates the proportion of variance in the effect sizes across studies that is explained by the meta-regression model. *n* indicates number of studies included. SE, standard error.

## Discussion

This meta-analysis establishes two important observations, i.e., that in individuals with circulating anti-Ro/SSA antibodies: (i) a longer QTc is present and (ii) a higher prevalence of QTc prolongation is also documented, when compared to those without. The validity of these findings is supported by the absence of publication bias in the overall analysis of continuous data studies focused on QTc values. Moreover, although evidence of publication bias was detected among binary studies assessing the incidence of QTc prolongation, possibly due to an underrepresentation of smaller studies reporting a lower incidence of QTc prolongation in anti-Ro/SSA-positive vs. -negative patients, meta-regression analysis of most included studies (13/15) revealed no association between ORs and study population size.

The above findings provide strong support for the observation that anti-Ro/SSA antibodies have a significant pathogenic role in promoting QTc prolongation. The result is particularly interesting because we observed a relatively pronounced increase in the frequency of QTc prolongation (more than two times), while the actual prolongation of QTc, though statistically significant, was not so pronounced. An explanation for this phenomenon may be found in figure 1 of the publication by Lazzerini *et al*.^7^ (see Supplementary material online, Appendix p. 33), a population study involving 7339 participants, making it sufficiently robust. This figure 1 shows that the peaks of both distributions (anti-Ro/SSA-positive and anti-Ro/SSA-negative individuals) are located at nearly the same point on the x-axis, approximately at 428.5 ms. This means the modes of both samples are almost identical, and this represents the most frequently observed QTc value. Beyond approximately 445 ms, it becomes apparent that anti-Ro/SSA-positive individuals begin to predominate, as would be expected hypothetically. This situation, where two groups have closely identical sample modes, but the anti-Ro/SSA-positive group starts to dominate at values >445 ms, could lead to a noticeably higher frequency of individuals with abnormally prolonged QTc in the anti-Ro/SSA-positive group, while the actual QTc values differ only modestly from the anti-Ro/SSA-negative group. The same pattern was observed in other study subgroups, i.e. unselected CTDs and SLE. In both groups, the increase in frequency of QTc prolongation was more pronounced than the actual difference in QTc values; even, in the SLE subgroup, the difference in QTc between anti-Ro/SSA-positive and anti-Ro/SSA-negative patients was not statistically significant. Overall, these findings suggest that although anti-Ro/SSA antibodies *per se* have a moderate, albeit significant, prolonging effect on the QTc, they can substantially contribute to promote the development of QTc prolongation, probably when other QT-prolonging risk factors are concomitantly present in a specific patient (multi-hit theory).^4,10^ Indeed, it is well recognized that a single risk factor alone, even including pathogenic mutations in LQTS genes,^41^ is often not able to induce LQTS, because many, often-redundant ion channel mechanisms are involved in preserving normal action potential duration in ventricles (repolarization reserve).^42^ However, in most of the available studies in this meta-analysis, no information is provided regarding the presence or not of concomitant QT-prolonging risk factors in anti-Ro/SSA-positive and negative patients. The only study in which such a potential issue was comprehensively analysed in a large population of over 7000 individuals, the fully adjusted multivariable analysis provided evidence that the risk of QTc prolongation >500 ms in anti-Ro/SSA-positive patients was approximately two times higher than in negative ones, although the absolute QTc difference between the two groups was only ∼6 ms.^7^

Current evidence coming from basic and clinical studies strongly suggests that only the anti-Ro/SSA-52kD antibody subtype can inhibit hERG and promote QTc prolongation.^8–10^ Indeed, although strictly associated at the molecular level to constitute the Ro-ribonucleoprotein, the 52-kD and 60-kD subunits are structurally very different,^5^ with the 52-kD component being the only one for which a significant sequence homology with the extracellular loop of hERG has been demonstrated.^8^ In addition, evidence indicates that the extent of anti-Ro/SSA-52kD-associated hERG-blocking effect is directly related to the autoantibody circulating levels, suggesting that a critical concentration threshold must be reached for being clinically manifest in terms of QTc prolongation.^8,16,19^ Because there are only five studies providing data on autoantibody subtypes, this meta-analysis could not objectively address the association between anti-Ro/SSA-52kD antibodies and QTc. Indeed, such a small number of studies was insufficient for a robust meta-analysis. Specifically, the available studies with binary outcomes are not enough to perform a sufficiently powered analysis and biased by disease-specific factors (e.g. inherent low anti-Ro/SSA-52kD levels in systemic sclerosis (SSc); see below in the ‘Meta-regression’ paragraph). To address this issue, more studies, also reporting specific data on circulating levels are needed to fully clarify the specific role of anti-Ro/SSA antibody subtypes on QTc.

Over the ≈25-year period encompassing the studies included in this meta-analysis, significant advancements occurred in both QTc measurement techniques and anti-Ro/SSA antibody detection methods. However, the meta-regression analysis revealed no significant association between publication year and either OR or SMD outcomes. An additional investigation examined whether study population size influenced effect estimates, as scalability differences across studies (ranging from dozens to hundreds of participants) might potentially affect results. The meta-regression demonstrated no significant relationship between sample size and either outcome measure. The only significant predictor identified was diagnosis category. Studies examining unselected CTDs showed significantly higher OR values compared to SLE-specific studies, though this association was not observed for SMD measures. The model (for OR) demonstrated successful explanation of heterogeneity (accounting for 32.5% of the original inconsistency), maintained a favorable study-to-predictor ratio, and showed acceptable residual heterogeneity (21%). Given the statistical significance (*P* = 0.025), this model holds practical value. This finding carries particular clinical importance as SLE currently dominates the literature investigating anti-Ro/SSA-associated QTc prolongation. The results suggest that among CTDs, SLE may not represent the disease in which anti-Ro/SSA-mediated QT-prolonging effects are more pronounced, warranting greater research attention to other CTDs. A possible explanation may be that, among different CTDs, a wide variability exists in terms of anti-Ro/SSA-52kD antibody presence and concentrations, both factors believed to be critically involved in determining the autoantibody-mediated electrophysiology interference leading to QTc prolongation.^8,9,16^ Specifically, in SLE, the prevalence and circulating levels of anti-Ro/SSA-52kD antibodies are significantly lower when compared to SS.^43^ Moreover, while this subtype is relatively common to find in SSc, in these patients, the antibody level is typically low.^44^ In agreement with these considerations, the only study specifically evaluating SSc patients did not find any association,^14^ while the only one focused on SS reported significantly longer QTc in anti-Ro/SSA-positive individuals.^36^ Indeed, such disease variability may account for the considerable heterogeneity (*I*^2^ = 68%) observed in studies including unselected CTDs patients.

This meta-analysis does not demonstrate a significant association between maternal anti-Ro/SSA antibodies and longer QTc in children. However, it should be noted that the included studies exhibited a high degree of heterogeneity (*I*^2^ = 81%). The small number of studies (*n* = 5) makes it difficult to identify the source of heterogeneity. Remarkably, the studies that reported an association between anti-Ro/SSA antibodies and increased QTc duration^20,22^ had very small control groups, both in absolute terms (seven subjects each) and relative to the experimental group (3- and 5-fold smaller, respectively). Although this observation could indirectly suggest that the remaining three studies with larger sample sizes^21,23,24^ may provide more reliable results, further studies are needed to reach any conclusion, also because none evaluated anti-Ro/SSA antibody subtypes (and related circulating levels). Moreover, evidence indicates that newborns may present a lower susceptibility to develop anti-Ro/SSA-associated QTc prolongation than adults (reduced calcium channel reserve in cardiomyocytes, in part offsetting the action potential duration-prolonging effect of autoantibody-induced hERG blockade),^45^ thereby possibly requiring larger sample sizes to detect the phenomenon in this kind of subjects. Indeed, although not providing data suitable for this meta-analysis, other studies found an association between maternal anti-Ro/SSA antibodies and transient QTc prolongation in newborns,^46,47^ consistent with the physiological clearance of every maternal-derived antibody during the first year of life. Furthermore, some cases of TdP occurring in anti-Ro/SSA-positive newborns have been also reported.^48^

This meta-analysis has some limitations. First, the number of available studies using a 460-ms or greater cut-off for QTc prolongation, as well as those examining the effects of specific antibody subtypes (anti-52kD/60kD) on QTc values and QTc prolongation prevalence is not sufficient for a robust statistical analysis. Moreover, some analyses, such as those evaluating the association between anti-Ro/SSA antibodies and QTc in infants, or between the anti-Ro/SSA-52kD subtype and the prevalence of QTc prolongation, show high study heterogeneity, making it difficult to conclusively confirm the reliability of the obtained results. Another possible limitation of the study is that most of the research involved predominantly female populations. However, given that meta-regression analysis did not find significant influence of sex, and considering the known sex-based heterogeneity in the prevalence of the target rheumatologic diseases, this limitation can be considered conditional. Similar considerations can be made for the concomitant use of QT-prolonging medications, specifically antimalarial drugs.

Another potential limitation is that the present meta-analysis does not include specific data on the percentage of anti-Ro/SSA-positive subjects presenting with a QTc prolongation >470 ms in men/480 ms in women [currently recognized as the most clinically relevant cut-off according to the American Heart Association (AHA)/American College of Cardiology (ACC) recommendations],^1^ nor on the occurrence of TdP or other arrhythmic events, because most of the included studies lack such information. Nevertheless, these aspects were specifically analysed by several single studies. In the largest study currently available, involving 7339 subjects (612 anti-Ro/SSA-positive),^7^ the authors found that a QTc prolongation >470 ms (men)/480 ms (women) was present in 10.0% of anti-Ro/SSA-positive patients (a value almost double compared to that observed in negative subjects, i.e. 6.2%; *P* < 0.001). Moreover, the same study also investigated the frequency of marked QTc prolongation, i.e. >500 ms (as defined by AHA/ACC recommendations, recognized to be a marker of significant TdP risk for both sexes)^1^ and found that it was approximately three times higher in anti-Ro/SSA-positive vs. -negative subjects (3.1% vs. 1.1%, *P* < 0.001).^7^

Moreover, another study included in the meta-analysis^29^ specifically analysed the relationship between anti-Ro/SSA positivity, QTc prolongation, and occurrence of ventricular arrhythmic events in unselected CTD patients, providing evidence that anti-Ro/SSA-positive patients had a higher incidence of complex VAs compared to the negative group (50% vs. 10%; *P* < 0.005), with a direct relation between global mean 24-h QTc and ventricular arrhythmic load, independently of age and disease duration.^29^ Consistently, a recent Israeli nationwide study involving >100 000 subjects (11 525 anti-Ro/SSA-positive) demonstrated that the presence of anti-Ro/SSA antibodies was associated with increased rates of paroxysmal ventricular tachycardia,^49^ i.e. a ∼50% higher risk in anti-Ro/SSA-positive vs. -negative subjects [OR = 1.49 (1.10–2.01), *P* = 0.009].^50^

Finally, although none of the studies included in the meta-analysis provided information on possible cases of TdP occurrence, it should be noted as one study^9^ (excluded from the meta-analysis due to inappropriate study design) found that in an unselected cohort of TdP patients, prospectively collected, regardless of concomitant diseases and ongoing medications, anti-Ro/SSA-52kD positivity was present in >50% of cases.^9^ Moreover, sera/IgG purified from anti-Ro/SSA-positive, but not negative, TdP patients significantly inhibited I_K_r and cross-reacted with the hERG-channel pore-forming region.^9^

## Conclusions

This meta-analysis supports the conclusion that circulating anti-Ro/SSA antibodies *per se* represent a significant risk factor for QTc prolongation (*Structured Graphical Abstract*). However, the presence of many studies with conflicting results suggests that a more accurate investigation of some specific variables, such as the impact of individual antibody subtype level (anti-52kD/anti-60kD) and the adjustment for concomitant QT-prolonging risk factors, will be critical to fully clarify the underlying mechanisms. Addressing this knowledge gap is expected to have significant clinical implications, as it may allow a more precise assessment of the arrhythmic risk in individual patients with circulating anti-Ro/SSA antibodies, thereby guiding targeted preventive strategies. They include receiving serial ECGs and specific counselling about the management of medications and other risk factors that could critically enhance their higher inherent susceptibility to TdP and other QT-related malignant arrhythmias.

## Supplementary Material

oeag123_Supplementary_Data

## Data Availability

All extracted data are provided either in the main manuscript or in the online supplement. No additional data are available

## Pre-registered clinical trial number

Study protocol was registered in PROSPERO (ID: CRD42024535293).
