# Supplementary material for: Circulating anti-Ro/Sjögren’s syndrome-related antigen A antibodies and risk of heart rate-corrected QT interval prolongation: a meta-analysis
Source: Eur Heart J Open. 2026 Aug 14;6(5):oeag123. doi: 10.1093/ehjopen/oeag123 (PMC13663800; doi:10.1093/ehjopen/oeag123)
Supplement: Supplementary file 1 [file oeag123_supplementary_data.pdf]

# SUPPLEMENTARY APPENDIX

## TABLE OF CONTENTS

SUPPLEMENTARY METHODS ........ 3
Study search ........ 3
SUPPLEMENTARY RESULTS ........ 5
Reports characteristics ........ 5
Cutoff values used in different studies ........ 5
Disease subgroups ........ 5
Appendix Table 1: Quality assessment of articles using The Joanna Briggs Institute (JBI) Critical Appraisal Checklist for analytical cross-sectional study ........ 6
Appendix Figure 1. Quality of studies assessment by STROBE statement. ........ 25
Appendix Figure 2. Risk of bias of each study included in meta-analyses assessed by RoBANS2. ........ 26
Appendix Figure 3. Contour-enhanced funnel plots. ........ 27
Appendix Figure 4. Contour-enhanced funnel plots for studies involving patients with unselected connective tissue diseases(CTD). ........ 28
Appendix Figure 5. Contour-enhanced funnel plots for studies involving patients with systemic lupus erythematosus(SLE). ........ 29
Appendix Figure 6. Contour-enhanced funnel plots for studies involving subjects in whom anti-Ro/SSA-52kD-antibody testing is available. ........ 30
Appendix Figure 7. Forest plots of the odds ratio of QTc prolongation in anti-Ro/SSA-60kD-positive vs. anti-Ro/SSA-60kD-negative individuals, regardless of the specific cutoff used. ........ 31
Appendix Figure 8. Contour-enhanced funnel plot for studies involving infants with anti-Ro/SSA positive mothers ........ 32
Appendix Figure 9. Empirical distribution of QTc values in a large cohort of 7,339 US veterans ........ 33
REFERENCES ........ 34

# SUPPLEMENTARY METHODS

## Study search

The following search query was used for PubMed and Google Scholar databases:

(Anti-Ro antibodies OR Anti-Ro/SSA antibodies OR Anti-Ro-52 kDa antibodies OR Anti-Ro-60 kDa antibodies AND QTc interval) OR (Anti-Ro antibodies OR Anti-Ro/SSA antibodies OR Anti-Ro-52 kDa antibodies OR Anti-Ro-60 kDa antibodies AND QT corrected) OR (Anti-Ro antibodies OR Anti-Ro/SSA antibodies OR Anti-Ro-52 kDa antibodies OR Anti-Ro-60 kDa antibodies AND QTc segment) OR (Anti-Ro antibodies OR Anti-Ro/SSA antibodies OR Anti-Ro-52 kDa antibodies OR Anti-Ro-60 kDa antibodies AND QTc prolongation) OR (Anti-Ro antibodies OR Anti-Ro/SSA antibodies OR Anti-Ro-52 kDa antibodies OR Anti-Ro-60 kDa antibodies AND QTc length) OR (Anti-Ro antibodies OR Anti-Ro/SSA antibodies OR Anti-Ro-52 kDa antibodies OR Anti-Ro-60 kDa antibodies AND ECG Anomalies) OR (Anti-Ro antibodies OR Anti-Ro/SSA antibodies OR Anti-Ro-52 kDa antibodies OR Anti-Ro-60 kDa antibodies AND Arrhythmia) OR (Anti-Ro antibodies OR Anti-Ro/SSA antibodies OR Anti-Ro-52 kDa antibodies OR Anti-Ro-60 kDa antibodies AND QT).

The following search query was used for Scopus database:

(Anti-Ro antibodies OR Anti-Ro/SSA antibodies OR Anti-Ro-52 kDa antibodies OR Anti-Ro-60 kDa antibodies AND QTc interval) OR (Anti-Ro antibodies OR Anti-Ro/SSA antibodies OR Anti-Ro-52 kDa antibodies OR Anti-Ro-60 kDa antibodies AND QTc segment) OR (Anti-Ro antibodies OR Anti-Ro/SSA antibodies OR Anti-Ro-52 kDa antibodies OR Anti-Ro-60 kDa antibodies AND QTc prolongation) OR (Anti-Ro antibodies OR Anti-Ro/SSA antibodies OR Anti-Ro-52 kDa antibodies OR Anti-Ro-60 kDa antibodies AND QTc length) OR (Anti-Ro antibodies OR Anti-Ro/SSA antibodies OR Anti-Ro-52 kDa antibodies OR Anti-Ro-60 kDa antibodies AND ECG Anomalies) OR (Anti-Ro antibodies OR Anti-Ro/SSA antibodies OR Anti-Ro-52 kDa antibodies OR Anti-Ro-

60 kDa antibodies AND Arrhythmia), since the Scopus search engine doesn’t allow to use two-letter query such as «QT» or «QT corrected».

The following search filters were applied (where possible): date from 1958 to 2025, human studies.

# SUPPLEMENTARY RESULTS

## Reports characteristics

Quality control check of the 25 studies included in the meta-analysis was performed using The Joanna Briggs Institute(JBI) Critical Appraisal Checklist for analytical cross-sectional studies (last amended in 2017) [1].

Detailed information on the results of the quality assurance review is available in **S-Table 1**.

## Cutoff values used in different studies

In one study, the cutoff was 430 ms [2]. In 9 studies, the cutoff for QTc was 440 ms[3–12]. In one study, the cutoff was considered to be 450 ms for men and 460 ms for women[13]. However, since no man in this study had a QTc duration ≥450 ms, this study was classified as one where the cutoff was 460 ms. In three studies, the cutoff was 460 ms[9, 13, 14] (second cutoff for[9]). In one study, the cutoff was 470 ms for men and 480 ms for women[15]. In one study, the cutoff was 500 ms[16].

## Disease subgroups

The two main disease subgroups on which the meta-analysis focused were unselected CTDs and SLE. Six studies provided results as binary data for unselected CTDs[3–5, 8, 12, 14]. Seven studies provided results as continuous data for unselected CTDs[3–5, 8, 12, 14, 17]. Seven studies provided results for SLE as binary data[2, 6, 9–11, 13, 16]. Continuous data for SLE were provided in five studies[9, 11, 13, 18, 19]. For the rest, ne study examined the general population, both sick and healthy, with or without anti-Ro/SSA-antibodies and their relationship with the length of the QTc[15]. One study included systemic sclerosis(SSc)[6], and another one SS [20].

## Appendix Table 1: Quality assessment of articles using The Joanna Briggs Institute (JBI) Critical Appraisal Checklist for analytical cross-sectional study

(last amended in 2017)[1]

| **Study 1: Gordon et al., (a) 2001** | | | |
|---|---|---|---|
| Major Components | Response options | | |
| 1. Were the criteria for inclusion in the sample clearly defined? | **Yes** | No | Unclear |
| 2. Were the study subjects and the setting described in detail? | **Yes** | No | Unclear |
| 3. Was the exposure measured in a valid and reliable way? | **Yes** | No | Unclear |
| 4. Were objective, standard criteria used for measurement of the condition? | **Yes** | No | Unclear |
| 5. Were confounding factors identified? | **Yes** | No | Unclear |
| 6. Were strategies to deal with confounding factors stated? | **Yes** | No | Unclear |
| 7. Were the outcomes measured in a valid and reliable way? | **Yes** | No | Unclear |
| 8. Was appropriate statistical analysis used? | **Yes** | No | Unclear |
| Overall appraisal: Include **Yes** Exclude ☐ Seek further info ☐ | | | |

| **Study 2: Lazzerini et al., 2004** | | | |
|---|---|---|---|
| Major Components | Response options | | |
| 1. Were the criteria for inclusion in the sample clearly defined? | **Yes** | No | Unclear |
| 2. Were the study subjects and the setting described in detail? | **Yes** | No | Unclear |
| 3. Was the exposure measured in a valid and reliable way? | **Yes** | No | Unclear |
| 4. Were objective, standard criteria used for measurement of the condition? | **Yes** | No | Unclear |
| 5. Were confounding factors identified? | **Yes** | No | Unclear |
| 6. Were strategies to deal with confounding factors stated? | **Yes** | No | Unclear |
| 7. Were the outcomes measured in a valid and reliable way? | **Yes** | No | Unclear |
| 8. Was appropriate statistical analysis used? | **Yes** | No | Unclear |
| Overall appraisal: Include **Yes** Exclude ☐ Seek further info ☐ | | | |

| **Study 3: Costedoat-Chalumeau et al., 2005** | | | |
|---|---|---|---|
| Major Components | Response options | | |
| 1. Were the criteria for inclusion in the sample clearly defined? | **Yes** | No | Unclear |
| 2. Were the study subjects and the setting described in detail? | **Yes** | No | Unclear |

| | | | |
|---|---|---|---|
| 3. Was the exposure measured in a valid and reliable way? | Yes | No | **Unclear** |
| 4. Were objective, standard criteria used for measurement of the condition? | **Yes** | No | Unclear |
| 5. Were confounding factors identified? | **Yes** | No | Unclear |
| 6. Were strategies to deal with confounding factors stated? | **Yes** | No | Unclear |
| 7. Were the outcomes measured in a valid and reliable way? | Yes | No | **Unclear** |
| 8. Was appropriate statistical analysis used? | **Yes** | No | Unclear |
| Overall appraisal: Include **Yes** Exclude ☐ Seek further info ☐ | | | |

| **Study 4: Cardoso et al., 2005** | | | |
|---|---|---|---|
| Major Components | Response options | | |
| 1. Were the criteria for inclusion in the sample clearly defined? | **Yes** | No | Unclear |
| 2. Were the study subjects and the setting described in detail? | **Yes** | No | Unclear |
| 3. Was the exposure measured in a valid and reliable way? | **Yes** | No | Unclear |
| 4. Were objective, standard criteria used for measurement of the condition? | **Yes** | No | Unclear |
| 5. Were confounding factors identified? | **Yes** | No | Unclear |
| 6. Were strategies to deal with confounding factors stated? | **Yes** | No | Unclear |
| 7. Were the outcomes measured in a valid and reliable way? | **Yes** | No | Unclear |

| | | | |
|---|---|---|---|
| 8. Was appropriate statistical analysis used? | **Yes** | No | Unclear |
| Overall appraisal: Include **Yes** Exclude ☐ Seek further info ☐ | | | |

| **Study 5: Lazzerini et al., 2007** | | | |
|---|---|---|---|
| Major Components | Response options | | |
| 1. Were the criteria for inclusion in the sample clearly defined? | **Yes** | No | Unclear |
| 2. Were the study subjects and the setting described in detail? | **Yes** | No | Unclear |
| 3. Was the exposure measured in a valid and reliable way? | **Yes** | No | Unclear |
| 4. Were objective, standard criteria used for measurement of the condition? | **Yes** | No | Unclear |
| 5. Were confounding factors identified? | **Yes** | No | Unclear |
| 6. Were strategies to deal with confounding factors stated? | **Yes** | No | Unclear |
| 7. Were the outcomes measured in a valid and reliable way? | **Yes** | No | Unclear |
| 8. Was appropriate statistical analysis used? | **Yes** | No | Unclear |
| Overall appraisal: Include **Yes** Exclude ☐ Seek further info ☐ | | | |

| **Study 6: Bourré-Tessier et al., 2011** |
|---|

| Major Components | Response options | | |
|---|---|---|---|
| 1. Were the criteria for inclusion in the sample clearly defined? | **Yes** | No | Unclear |
| 2. Were the study subjects and the setting described in detail? | **Yes** | No | Unclear |
| 3. Was the exposure measured in a valid and reliable way? | **Yes** | No | Unclear |
| 4. Were objective, standard criteria used for measurement of the condition? | **Yes** | No | Unclear |
| 5. Were confounding factors identified? | **Yes** | No | Unclear |
| 6. Were strategies to deal with confounding factors stated? | **Yes** | No | Unclear |
| 7. Were the outcomes measured in a valid and reliable way? | **Yes** | No | Unclear |
| 8. Was appropriate statistical analysis used? | **Yes** | No | Unclear |
| Overall appraisal: Include **Yes** Exclude ☐ Seek further info ☐ | | | |

| **Study 7: Lazzerini et al., 2011** | | | |
|---|---|---|---|
| Major Components | Response options | | |
| 1. Were the criteria for inclusion in the sample clearly defined? | **Yes** | No | Unclear |
| 2. Were the study subjects and the setting described in detail? | **Yes** | No | Unclear |
| 3. Was the exposure measured in a valid and reliable way? | **Yes** | No | Unclear |
| 4. Were objective, standard criteria used for measurement of the condition? | **Yes** | No | Unclear |

| | | | |
|---|---|---|---|
| 5. Were confounding factors identified? | **Yes** | No | Unclear |
| 6. Were strategies to deal with confounding factors stated? | **Yes** | No | Unclear |
| 7. Were the outcomes measured in a valid and reliable way? | **Yes** | No | Unclear |
| 8. Was appropriate statistical analysis used? | **Yes** | No | Unclear |
| Overall appraisal: Include **Yes** Exclude ☐ Seek further info ☐ | | | |

| **Study 8: Nomura et al., 2014** | | | |
|---|---|---|---|
| Major Components | Response options | | |
| 1. Were the criteria for inclusion in the sample clearly defined? | **Yes** | No | Unclear |
| 2. Were the study subjects and the setting described in detail? | **Yes** | No | Unclear |
| 3. Was the exposure measured in a valid and reliable way? | **Yes** | No | Unclear |
| 4. Were objective, standard criteria used for measurement of the condition? | **Yes** | No | Unclear |
| 5. Were confounding factors identified? | **Yes** | No | Unclear |
| 6. Were strategies to deal with confounding factors stated? | **Yes** | No | Unclear |
| 7. Were the outcomes measured in a valid and reliable way? | **Yes** | No | Unclear |
| 8. Was appropriate statistical analysis used? | **Yes** | No | Unclear |

| |
|---|
| Overall appraisal: Include **Yes** Exclude ☐ Seek further info ☐ |

| **Study 9: Teixeira et al., 2014** | | | |
|---|---|---|---|
| Major Components | Response options | | |
| 1. Were the criteria for inclusion in the sample clearly defined? | **Yes** | No | Unclear |
| 2. Were the study subjects and the setting described in detail? | **Yes** | No | Unclear |
| 3. Was the exposure measured in a valid and reliable way? | **Yes** | No | Unclear |
| 4. Were objective, standard criteria used for measurement of the condition? | **Yes** | No | Unclear |
| 5. Were confounding factors identified? | **Yes** | No | Unclear |
| 6. Were strategies to deal with confounding factors stated? | **Yes** | No | Unclear |
| 7. Were the outcomes measured in a valid and reliable way? | **Yes** | No | Unclear |
| 8. Was appropriate statistical analysis used? | **Yes** | No | Unclear |
| Overall appraisal: Include **Yes** Exclude ☐ Seek further info ☐ | | | |

| **Study 10: Massie et al., 2014** | |
|---|---|
| Major Components | Response options |

| | | | |
|---|---|---|---|
| 1. Were the criteria for inclusion in the sample clearly defined? | **Yes** | No | Unclear |
| 2. Were the study subjects and the setting described in detail? | **Yes** | No | Unclear |
| 3. Was the exposure measured in a valid and reliable way? | **Yes** | No | Unclear |
| 4. Were objective, standard criteria used for measurement of the condition? | **Yes** | No | Unclear |
| 5. Were confounding factors identified? | **Yes** | No | Unclear |
| 6. Were strategies to deal with confounding factors stated? | **Yes** | No | Unclear |
| 7. Were the outcomes measured in a valid and reliable way? | **Yes** | No | Unclear |
| 8. Was appropriate statistical analysis used? | **Yes** | No | Unclear |
| Overall appraisal: Include **Yes** Exclude ☐ Seek further info ☐ | | | |

| **Study 11: Pisoni et al., (a) 2015** | | | |
|---|---|---|---|
| Major Components | Response options | | |
| 1. Were the criteria for inclusion in the sample clearly defined? | **Yes** | No | Unclear |
| 2. Were the study subjects and the setting described in detail? | **Yes** | No | Unclear |
| 3. Was the exposure measured in a valid and reliable way? | **Yes** | No | Unclear |
| 4. Were objective, standard criteria used for measurement of the condition? | **Yes** | No | Unclear |
| 5. Were confounding factors identified? | **Yes** | No | Unclear |

| | | | |
|---|---|---|---|
| 6. Were strategies to deal with confounding factors stated? | **Yes** | No | Unclear |
| 7. Were the outcomes measured in a valid and reliable way? | **Yes** | No | Unclear |
| 8. Was appropriate statistical analysis used? | **Yes** | No | Unclear |
| Overall appraisal: Include **Yes** Exclude ☐ Seek further info ☐ | | | |

| **Study 12: Bourré-Tessier et al., 2015** | | | |
|---|---|---|---|
| Major Components | Response options | | |
| 1. Were the criteria for inclusion in the sample clearly defined? | **Yes** | No | Unclear |
| 2. Were the study subjects and the setting described in detail? | **Yes** | No | Unclear |
| 3. Was the exposure measured in a valid and reliable way? | **Yes** | No | Unclear |
| 4. Were objective, standard criteria used for measurement of the condition? | **Yes** | No | Unclear |
| 5. Were confounding factors identified? | **Yes** | No | Unclear |
| 6. Were strategies to deal with confounding factors stated? | **Yes** | No | Unclear |
| 7. Were the outcomes measured in a valid and reliable way? | **Yes** | No | Unclear |
| 8. Was appropriate statistical analysis used? | **Yes** | No | Unclear |
| Overall appraisal: Include **Yes** Exclude ☐ Seek further info ☐ | | | |

| **Study 13: Tufan et al., (a) 2017** | | | |
|---|---|---|---|
| Major Components | Response options | | |
| 1. Were the criteria for inclusion in the sample clearly defined? | **Yes** | No | Unclear |
| 2. Were the study subjects and the setting described in detail? | **Yes** | No | Unclear |
| 3. Was the exposure measured in a valid and reliable way? | **Yes** | No | Unclear |
| 4. Were objective, standard criteria used for measurement of the condition? | **Yes** | No | Unclear |
| 5. Were confounding factors identified? | **Yes** | No | Unclear |
| 6. Were strategies to deal with confounding factors stated? | **Yes** | No | Unclear |
| 7. Were the outcomes measured in a valid and reliable way? | **Yes** | No | Unclear |
| 8. Was appropriate statistical analysis used? | **Yes** | No | Unclear |
| Overall appraisal: Include **Yes** Exclude ☐ Seek further info ☐ | | | |

| **Study 14: Mostafavi et al., 2020** | | | |
|---|---|---|---|
| Major Components | Response options | | |
| 1. Were the criteria for inclusion in the sample clearly defined? | **Yes** | No | Unclear |
| 2. Were the study subjects and the setting described in detail? | **Yes** | No | Unclear |

| | | | |
|---|---|---|---|
| 3. Was the exposure measured in a valid and reliable way? | **Yes** | No | Unclear |
| 4. Were objective, standard criteria used for measurement of the condition? | **Yes** | No | Unclear |
| 5. Were confounding factors identified? | **Yes** | No | Unclear |
| 6. Were strategies to deal with confounding factors stated? | Yes | **No** | Unclear |
| 7. Were the outcomes measured in a valid and reliable way? | **Yes** | No | Unclear |
| 8. Was appropriate statistical analysis used? | **Yes** | No | Unclear |
| Overall appraisal: Include **Yes** Exclude ☐ Seek further info ☐ | | | |

| **Study 15: Azharudeen et al., (a) 2020** | | | |
|---|---|---|---|
| Major Components | Response options | | |
| 1. Were the criteria for inclusion in the sample clearly defined? | **Yes** | No | Unclear |
| 2. Were the study subjects and the setting described in detail? | **Yes** | No | Unclear |
| 3. Was the exposure measured in a valid and reliable way? | **Yes** | No | Unclear |
| 4. Were objective, standard criteria used for measurement of the condition? | **Yes** | No | Unclear |
| 5. Were confounding factors identified? | **Yes** | No | Unclear |
| 6. Were strategies to deal with confounding factors stated? | **Yes** | No | Unclear |
| 7. Were the outcomes measured in a valid and reliable way? | **Yes** | No | Unclear |

| | | | |
|---|---|---|---|
| 8. Was appropriate statistical analysis used? | **Yes** | No | Unclear |
| Overall appraisal: Include **Yes** Exclude ☐ Seek further info ☐ | | | |

| **Study 16: Villuendas et al., (a) 2021** | | | |
|---|---|---|---|
| Major Components | Response options | | |
| 1. Were the criteria for inclusion in the sample clearly defined? | **Yes** | No | Unclear |
| 2. Were the study subjects and the setting described in detail? | **Yes** | No | Unclear |
| 3. Was the exposure measured in a valid and reliable way? | **Yes** | No | Unclear |
| 4. Were objective, standard criteria used for measurement of the condition? | **Yes** | No | Unclear |
| 5. Were confounding factors identified? | **Yes** | No | Unclear |
| 6. Were strategies to deal with confounding factors stated? | **Yes** | No | Unclear |
| 7. Were the outcomes measured in a valid and reliable way? | **Yes** | No | Unclear |
| 8. Was appropriate statistical analysis used? | **Yes** | No | Unclear |
| Overall appraisal: Include **Yes** Exclude ☐ Seek further info ☐ | | | |

| **Study 17: Lazzerini et al., (a) 2021** |
|---|

| Major Components | Response options | | |
|---|---|---|---|
| 1. Were the criteria for inclusion in the sample clearly defined? | **Yes** | No | Unclear |
| 2. Were the study subjects and the setting described in detail? | **Yes** | No | Unclear |
| 3. Was the exposure measured in a valid and reliable way? | **Yes** | No | Unclear |
| 4. Were objective, standard criteria used for measurement of the condition? | **Yes** | No | Unclear |
| 5. Were confounding factors identified? | **Yes** | No | Unclear |
| 6. Were strategies to deal with confounding factors stated? | **Yes** | No | Unclear |
| 7. Were the outcomes measured in a valid and reliable way? | **Yes** | No | Unclear |
| 8. Was appropriate statistical analysis used? | **Yes** | No | Unclear |
| Overall appraisal: Include **Yes** Exclude ☐ Seek further info ☐ | | | |

| **Study 18: Gamazo-Herrero et al., (a) 2024** | | | |
|---|---|---|---|
| Major Components | Response options | | |
| 1. Were the criteria for inclusion in the sample clearly defined? | **Yes** | No | Unclear |
| 2. Were the study subjects and the setting described in detail? | **Yes** | No | Unclear |
| 3. Was the exposure measured in a valid and reliable way? | **Yes** | No | Unclear |
| 4. Were objective, standard criteria used for measurement of the condition? | **Yes** | No | Unclear |

| | | | |
|---|---|---|---|
| 5. Were confounding factors identified? | **Yes** | No | Unclear |
| 6. Were strategies to deal with confounding factors stated? | **Yes** | No | Unclear |
| 7. Were the outcomes measured in a valid and reliable way? | **Yes** | No | Unclear |
| 8. Was appropriate statistical analysis used? | **Yes** | No | Unclear |
| Overall appraisal: Include **Yes** Exclude ☐ Seek further info ☐ | | | |

| **Study 19: Wang et al., (a) 2024** | | | |
|---|---|---|---|
| Major Components | Response options | | |
| 1. Were the criteria for inclusion in the sample clearly defined? | **Yes** | No | Unclear |
| 2. Were the study subjects and the setting described in detail? | **Yes** | No | Unclear |
| 3. Was the exposure measured in a valid and reliable way? | **Yes** | No | Unclear |
| 4. Were objective, standard criteria used for measurement of the condition? | **Yes** | No | Unclear |
| 5. Were confounding factors identified? | **Yes** | No | Unclear |
| 6. Were strategies to deal with confounding factors stated? | **Yes** | No | Unclear |
| 7. Were the outcomes measured in a valid and reliable way? | **Yes** | No | Unclear |
| 8. Was appropriate statistical analysis used? | **Yes** | No | Unclear |

| Overall appraisal: Include **Yes** Exclude ☐ Seek further info ☐ |
|---|

| **Study 20: Ösken et al., 2025** | | | |
|---|---|---|---|
| Major Components | Response options | | |
| 1. Were the criteria for inclusion in the sample clearly defined? | **Yes** | No | Unclear |
| 2. Were the study subjects and the setting described in detail? | **Yes** | No | Unclear |
| 3. Was the exposure measured in a valid and reliable way? | **Yes** | No | Unclear |
| 4. Were objective, standard criteria used for measurement of the condition? | **Yes** | No | Unclear |
| 5. Were confounding factors identified? | **Yes** | No | Unclear |
| 6. Were strategies to deal with confounding factors stated? | **Yes** | No | Unclear |
| 7. Were the outcomes measured in a valid and reliable way? | **Yes** | No | Unclear |
| 8. Was appropriate statistical analysis used? | **Yes** | No | Unclear |
| Overall appraisal: Include **Yes** Exclude ☐ Seek further info ☐ | | | |

| **Study 21: Cimaz et al., 2001** |
|---|

| Major Components | Response options | | |
|---|---|---|---|
| 1. Were the criteria for inclusion in the sample clearly defined? | **Yes** | No | Unclear |
| 2. Were the study subjects and the setting described in detail? | **Yes** | No | Unclear |
| 3. Was the exposure measured in a valid and reliable way? | **Yes** | No | Unclear |
| 4. Were objective, standard criteria used for measurement of the condition? | **Yes** | No | Unclear |
| 5. Were confounding factors identified? | **Yes** | No | Unclear |
| 6. Were strategies to deal with confounding factors stated? | **Yes** | No | Unclear |
| 7. Were the outcomes measured in a valid and reliable way? | **Yes** | No | Unclear |
| 8. Was appropriate statistical analysis used? | **Yes** | No | Unclear |
| Overall appraisal: Include **Yes** Exclude ☐ Seek further info ☐ | | | |

| **Study 22: Gordon et al., (b) 2001** | | | |
|---|---|---|---|
| Major Components | Response options | | |
| 1. Were the criteria for inclusion in the sample clearly defined? | **Yes** | No | Unclear |
| 2. Were the study subjects and the setting described in detail? | **Yes** | No | Unclear |
| 3. Was the exposure measured in a valid and reliable way? | **Yes** | No | Unclear |

| | | | |
|---|---|---|---|
| 4. Were objective, standard criteria used for measurement of the condition? | **Yes** | No | Unclear |
| 5. Were confounding factors identified? | **Yes** | No | Unclear |
| 6. Were strategies to deal with confounding factors stated? | **Yes** | No | Unclear |
| 7. Were the outcomes measured in a valid and reliable way? | **Yes** | No | Unclear |
| 8. Was appropriate statistical analysis used? | **Yes** | No | Unclear |
| Overall appraisal: Include **Yes** Exclude ☐ Seek further info ☐ | | | |

| **Study 23: Costedoat-Chalumeau et al. et al., 2004** | | | |
|---|---|---|---|
| Major Components | Response options | | |
| 1. Were the criteria for inclusion in the sample clearly defined? | **Yes** | No | Unclear |
| 2. Were the study subjects and the setting described in detail? | **Yes** | No | Unclear |
| 3. Was the exposure measured in a valid and reliable way? | **Yes** | No | Unclear |
| 4. Were objective, standard criteria used for measurement of the condition? | **Yes** | No | Unclear |
| 5. Were confounding factors identified? | **Yes** | No | Unclear |
| 6. Were strategies to deal with confounding factors stated? | **Yes** | No | Unclear |
| 7. Were the outcomes measured in a valid and reliable way? | **Yes** | No | Unclear |
| 8. Was appropriate statistical analysis used? | **Yes** | No | Unclear |

| |
|---|
| Overall appraisal: Include **Yes** Exclude ☐ Seek further info ☐ |

| **Study 24: Gerosa et al., 2007** | | | |
|---|---|---|---|
| Major Components | Response options | | |
| 1. Were the criteria for inclusion in the sample clearly defined? | **Yes** | No | Unclear |
| 2. Were the study subjects and the setting described in detail? | **Yes** | No | Unclear |
| 3. Was the exposure measured in a valid and reliable way? | **Yes** | No | Unclear |
| 4. Were objective, standard criteria used for measurement of the condition? | **Yes** | No | Unclear |
| 5. Were confounding factors identified? | **Yes** | No | Unclear |
| 6. Were strategies to deal with confounding factors stated? | **Yes** | No | Unclear |
| 7. Were the outcomes measured in a valid and reliable way? | **Yes** | No | Unclear |
| 8. Was appropriate statistical analysis used? | **Yes** | No | Unclear |
| Overall appraisal: Include **Yes** Exclude ☐ Seek further info ☐ | | | |

| **Study 25: Motta et al., 2007** | |
|---|---|
| Major Components | Response options |

| | | | |
|---|---|---|---|
| 1. Were the criteria for inclusion in the sample clearly defined? | **Yes** | No | Unclear |
| 2. Were the study subjects and the setting described in detail? | **Yes** | No | Unclear |
| 3. Was the exposure measured in a valid and reliable way? | **Yes** | No | Unclear |
| 4. Were objective, standard criteria used for measurement of the condition? | **Yes** | No | Unclear |
| 5. Were confounding factors identified? | **Yes** | No | Unclear |
| 6. Were strategies to deal with confounding factors stated? | **Yes** | No | Unclear |
| 7. Were the outcomes measured in a valid and reliable way? | **Yes** | No | Unclear |
| 8. Was appropriate statistical analysis used? | **Yes** | No | Unclear |
| Overall appraisal: Include **Yes** Exclude ☐ Seek further info ☐ | | | |

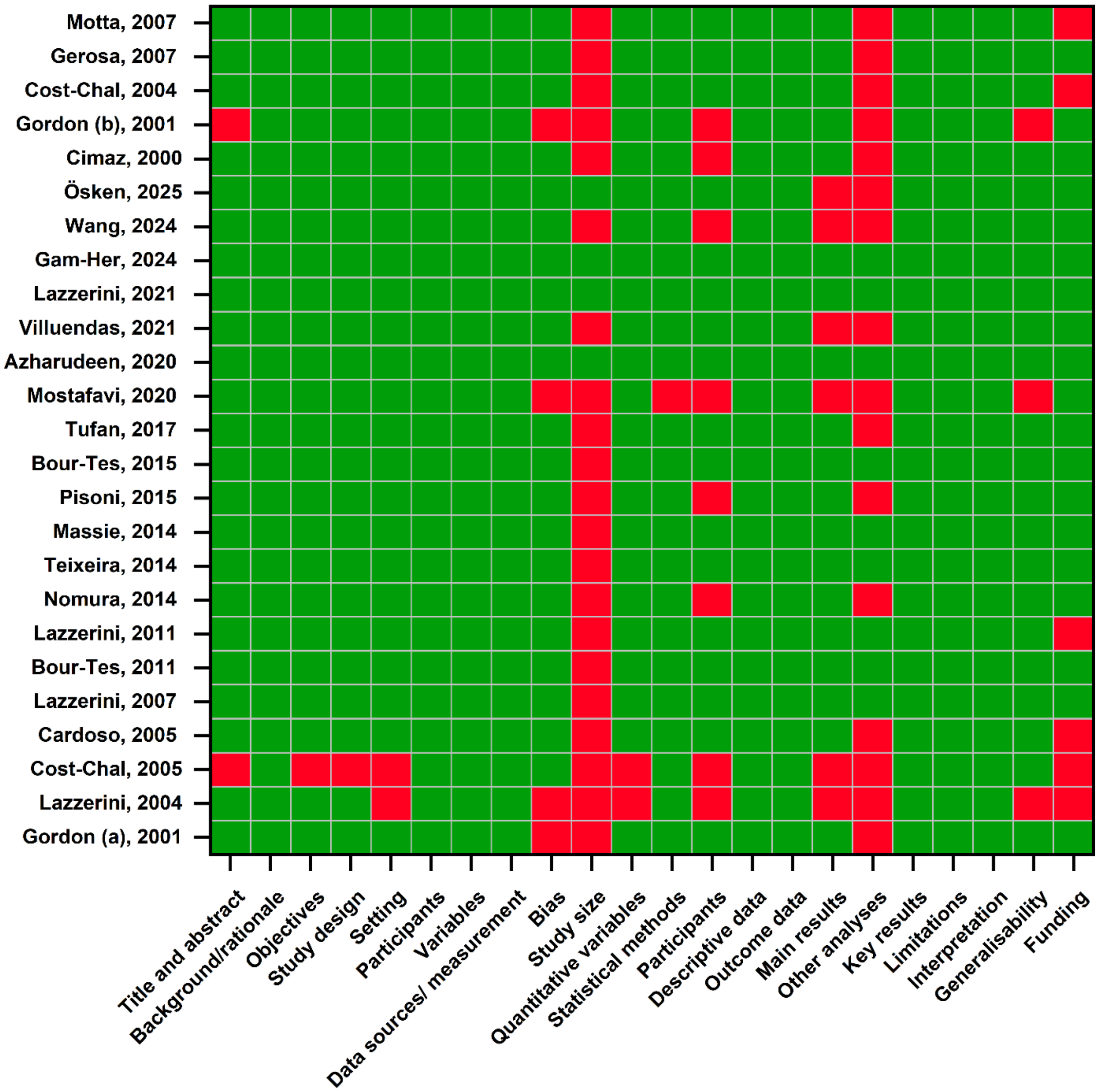


## Appendix Figure 1. Quality of studies assessment by STROBE statement.

A green square indicates that the study passes the test for this criterion. A red square indicates that the study does not pass the test for this criterion.

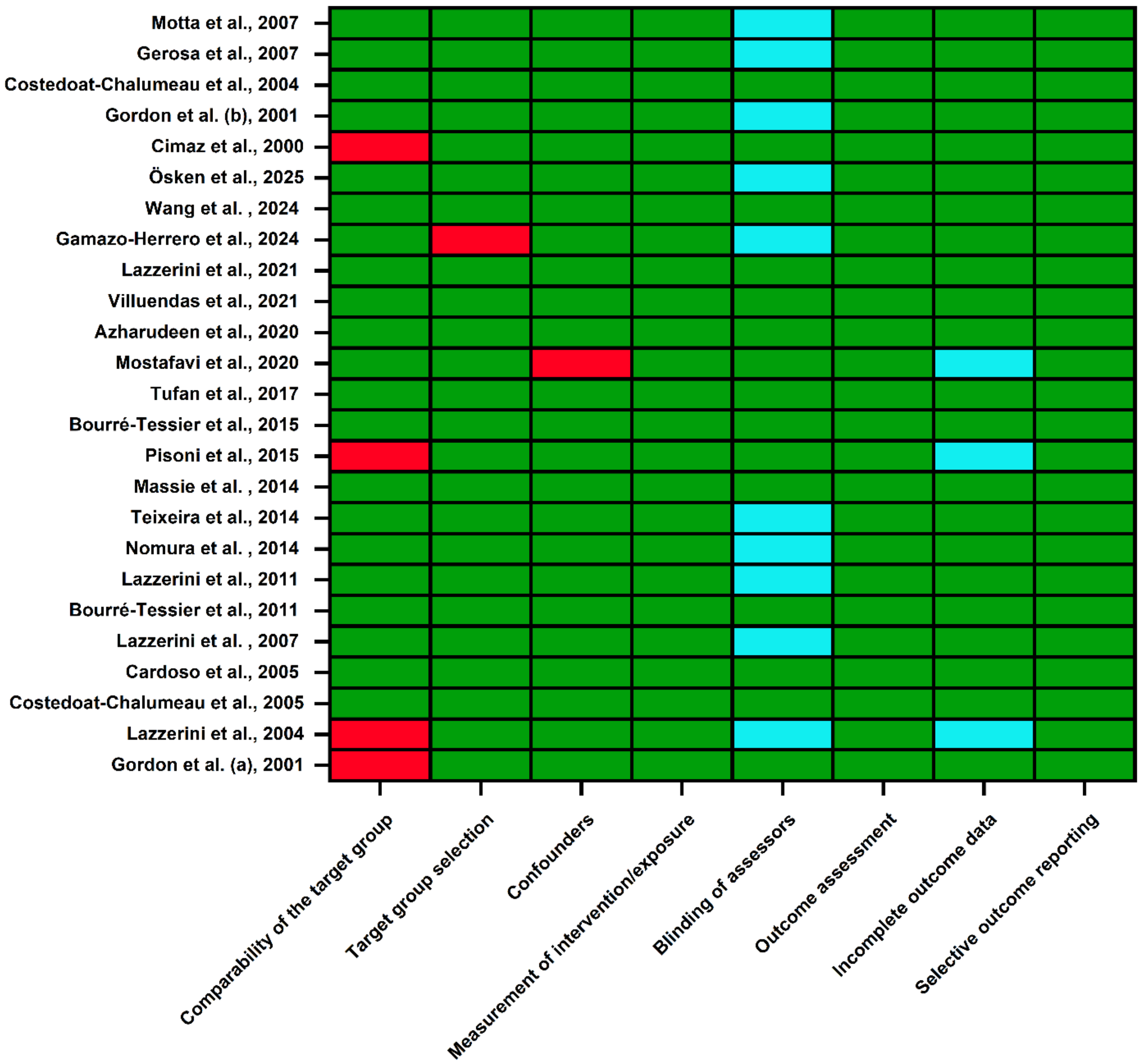


## Appendix Figure 2. Risk of bias of each study included in meta-analyses assessed by RoBANS2.

Green rectangles indicate a low risk of bias, cyan rectangles indicate an unclear risk of bias, red rectangles indicate a high risk of study bias for a particular criterion.

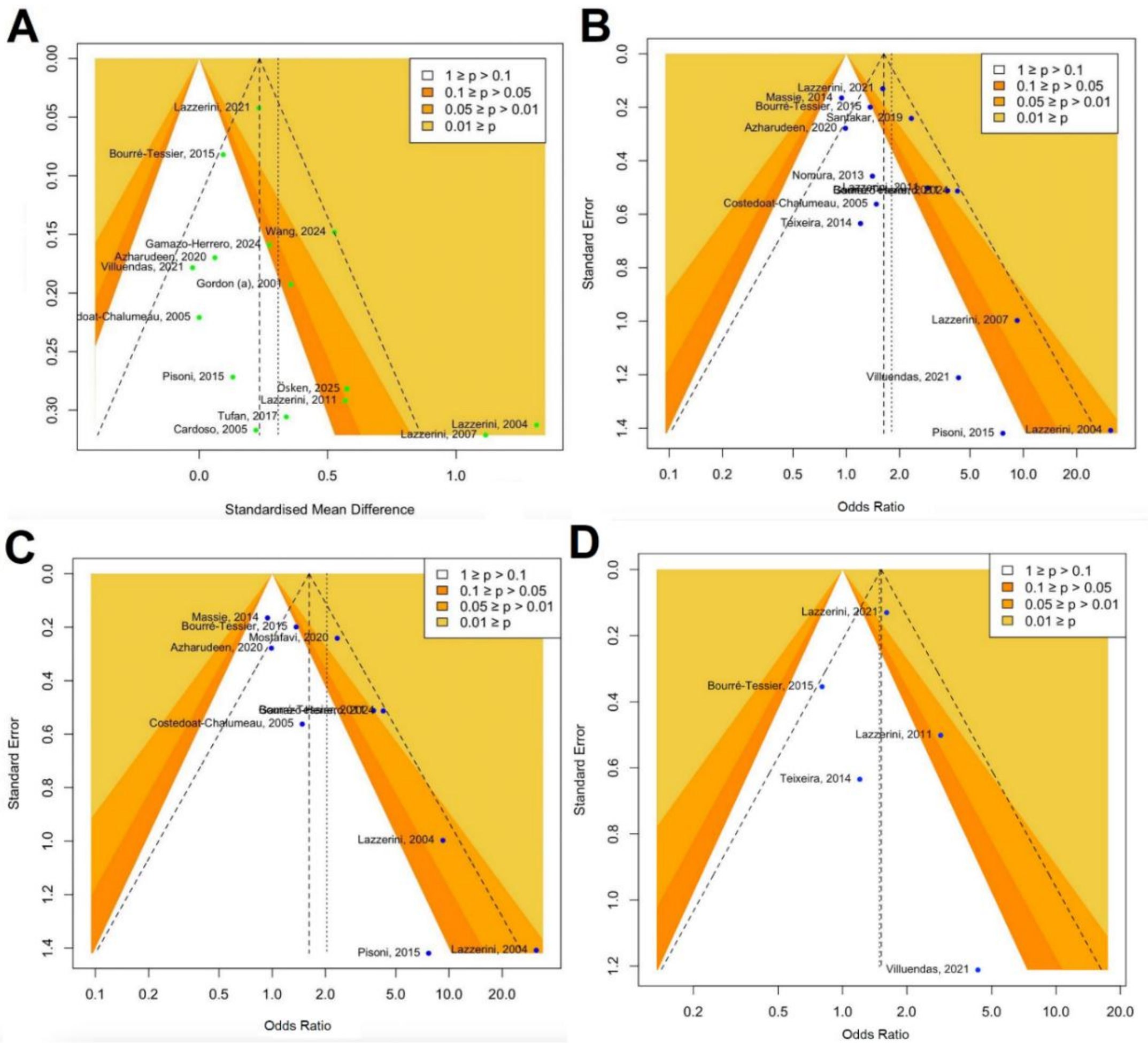

## Appendix Figure 3. Contour-enhanced funnel plots.

A) continuous data outcomes for QTc duration; B) binary data outcomes for QTc prolongation, regardless of the specific cutoff used; C) binary data outcomes for QTc prolongation with a cutoff of 440 ms; D) binary data outcomes for QTc prolongation with a cutoff of 460 ms or greater.

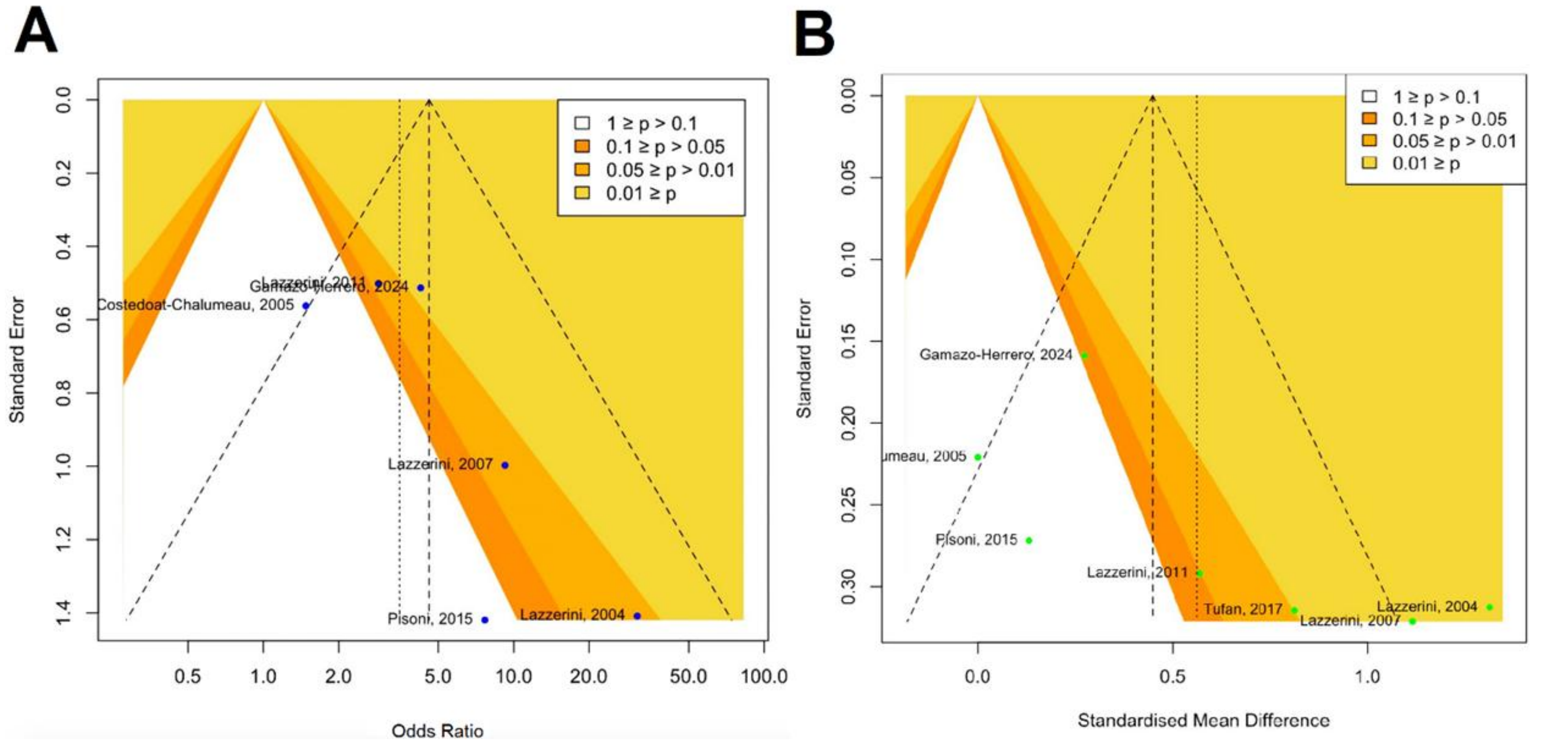


Appendix Figure 4. Contour-enhanced funnel plots for studies involving patients with unselected connective tissue diseases(CTD).

A) binary data outcomes; B) continuous data outcomes.

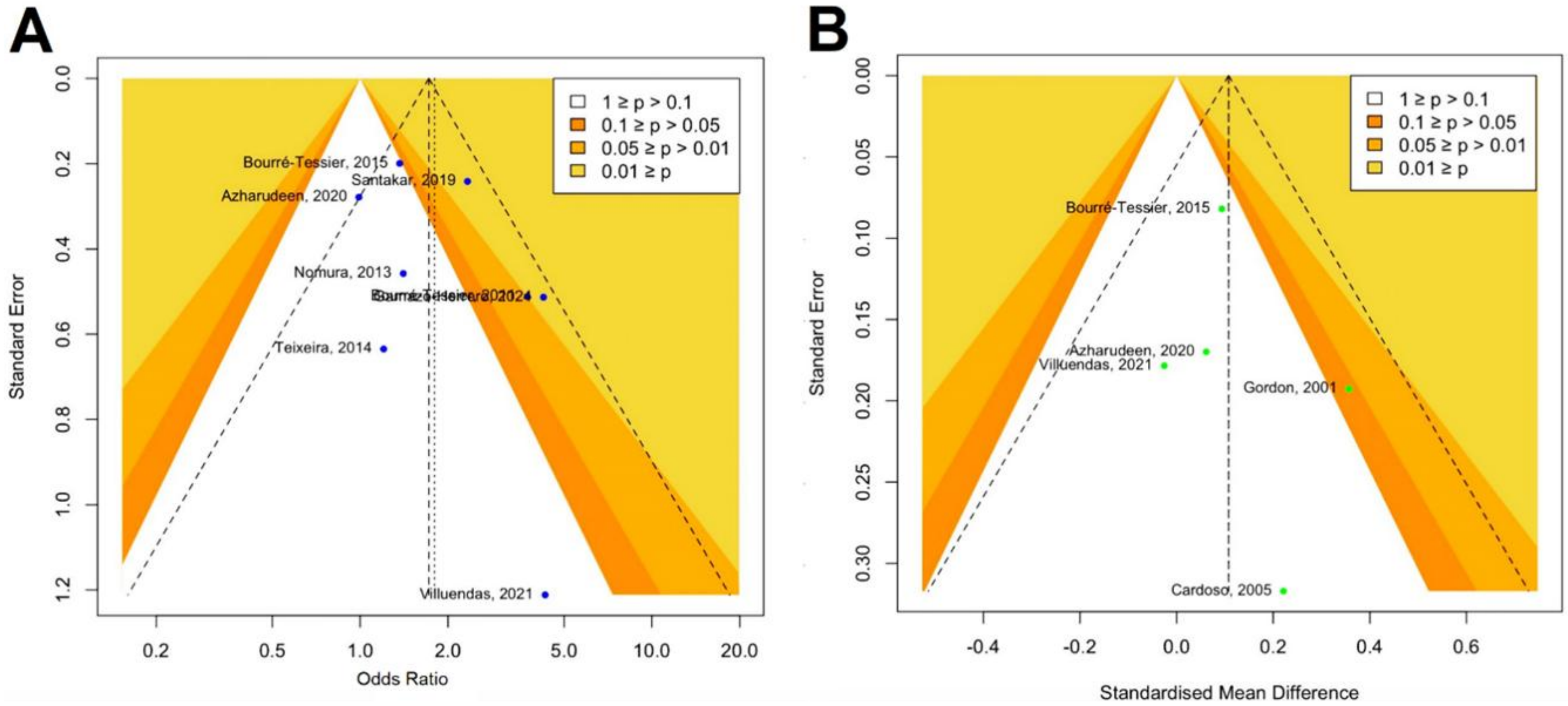


Appendix Figure 5. Contour-enhanced funnel plots for studies involving patients with systemic lupus erythematosus(SLE).

A) binary data outcomes; B) continuous data outcomes.

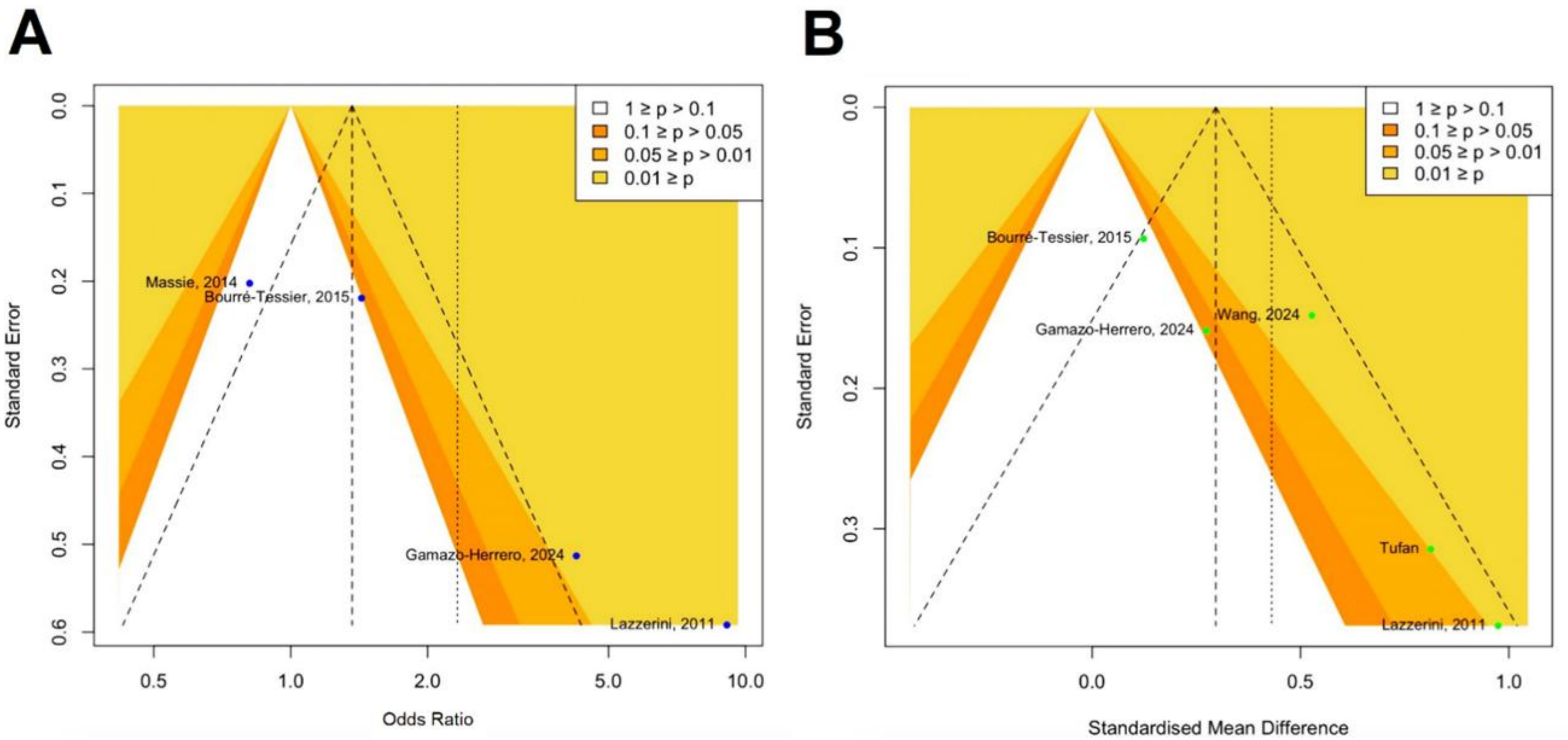


Appendix Figure 6. Contour-enhanced funnel plots for studies involving subjects in whom anti-Ro/SSA-52kD-antibody testing is available.

A) binary data outcomes; B) continuous data outcomes.

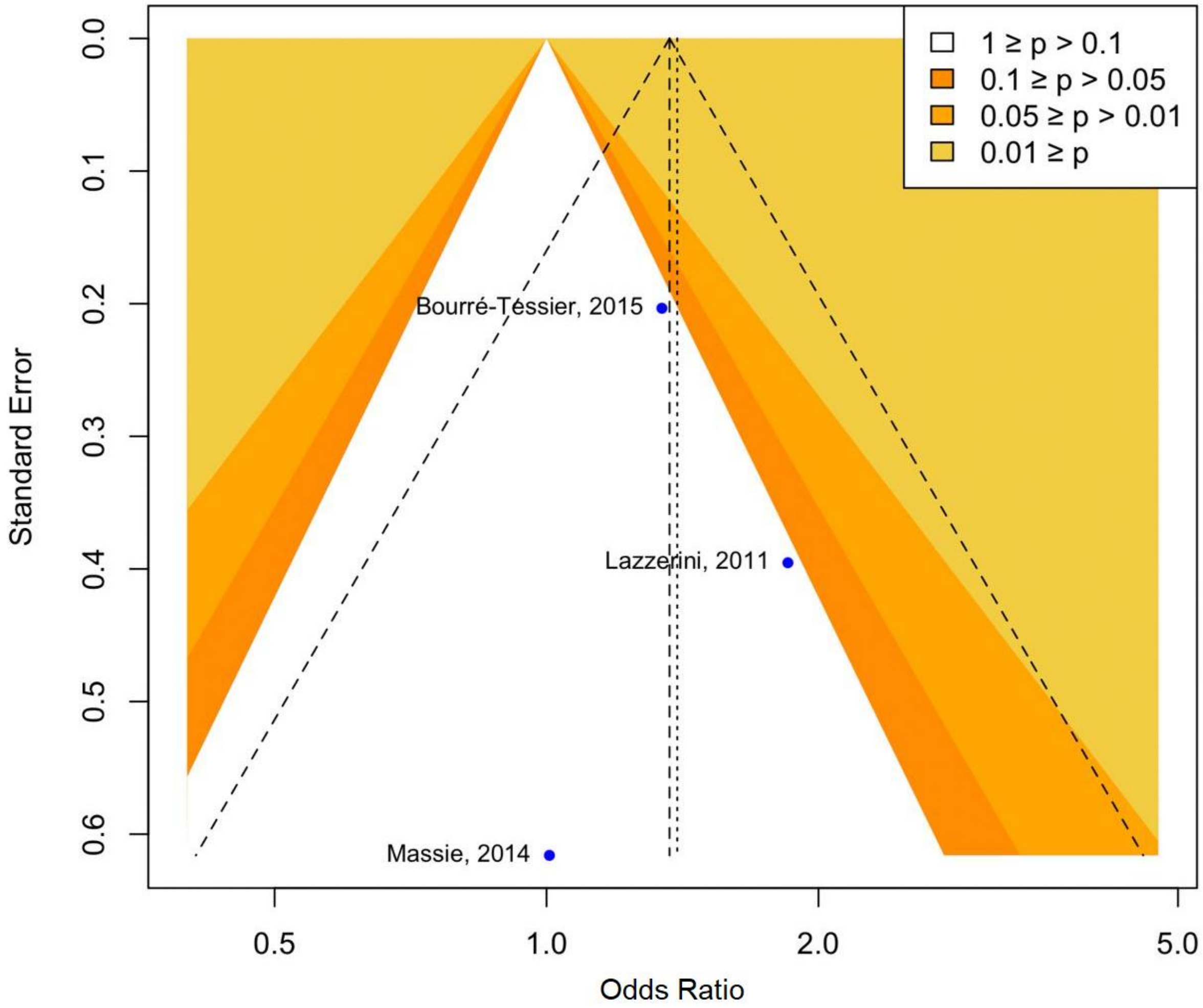


Appendix Figure 7. Forest plots of the odds ratio of QTc prolongation in anti-Ro/SSA-60kD-positive vs. anti-Ro/SSA-60kD-negative individuals, regardless of the specific cutoff used.

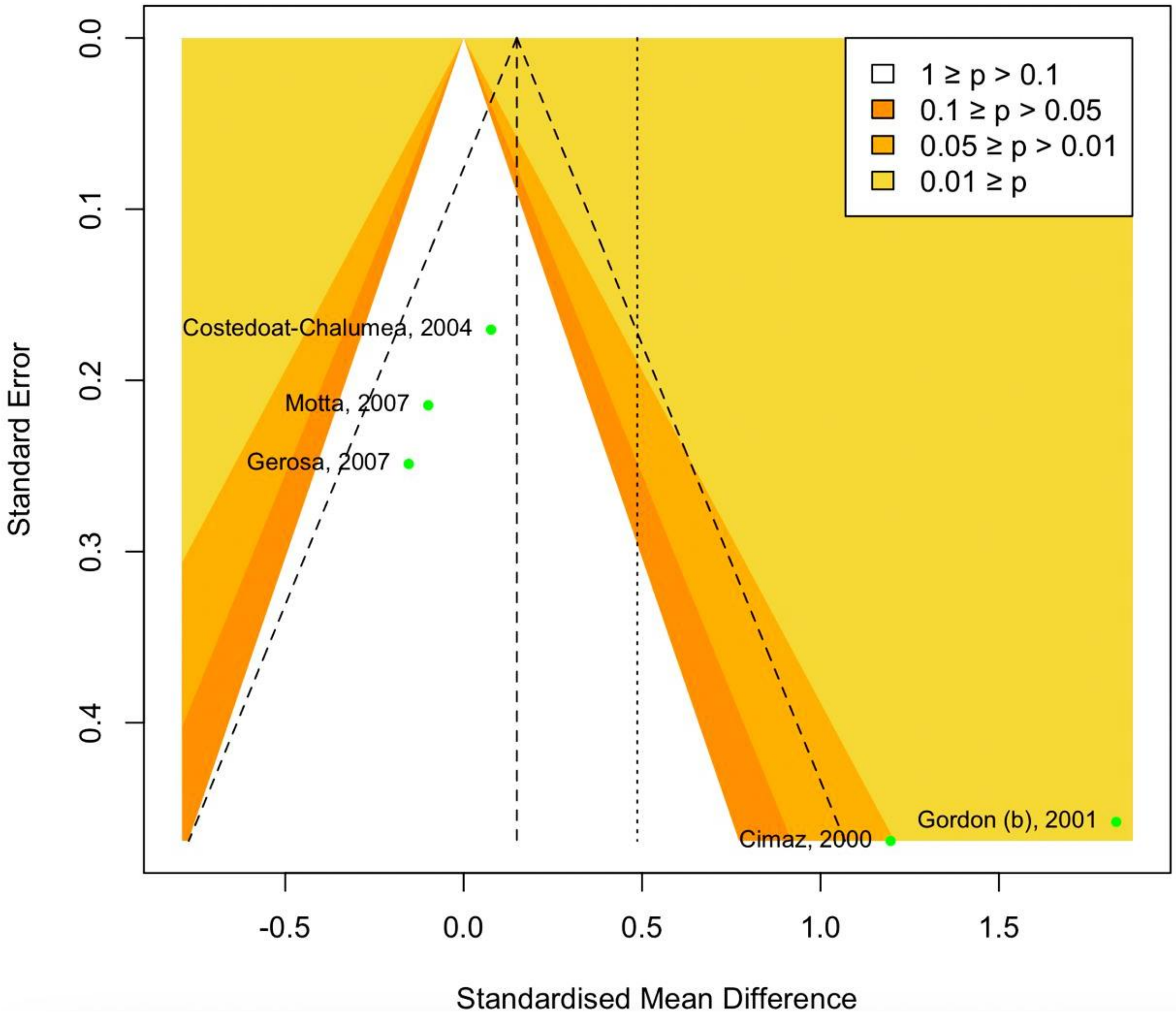


Appendix Figure 8. Contour-enhanced funnel plot for studies involving infants with anti-Ro/SSA positive mothers

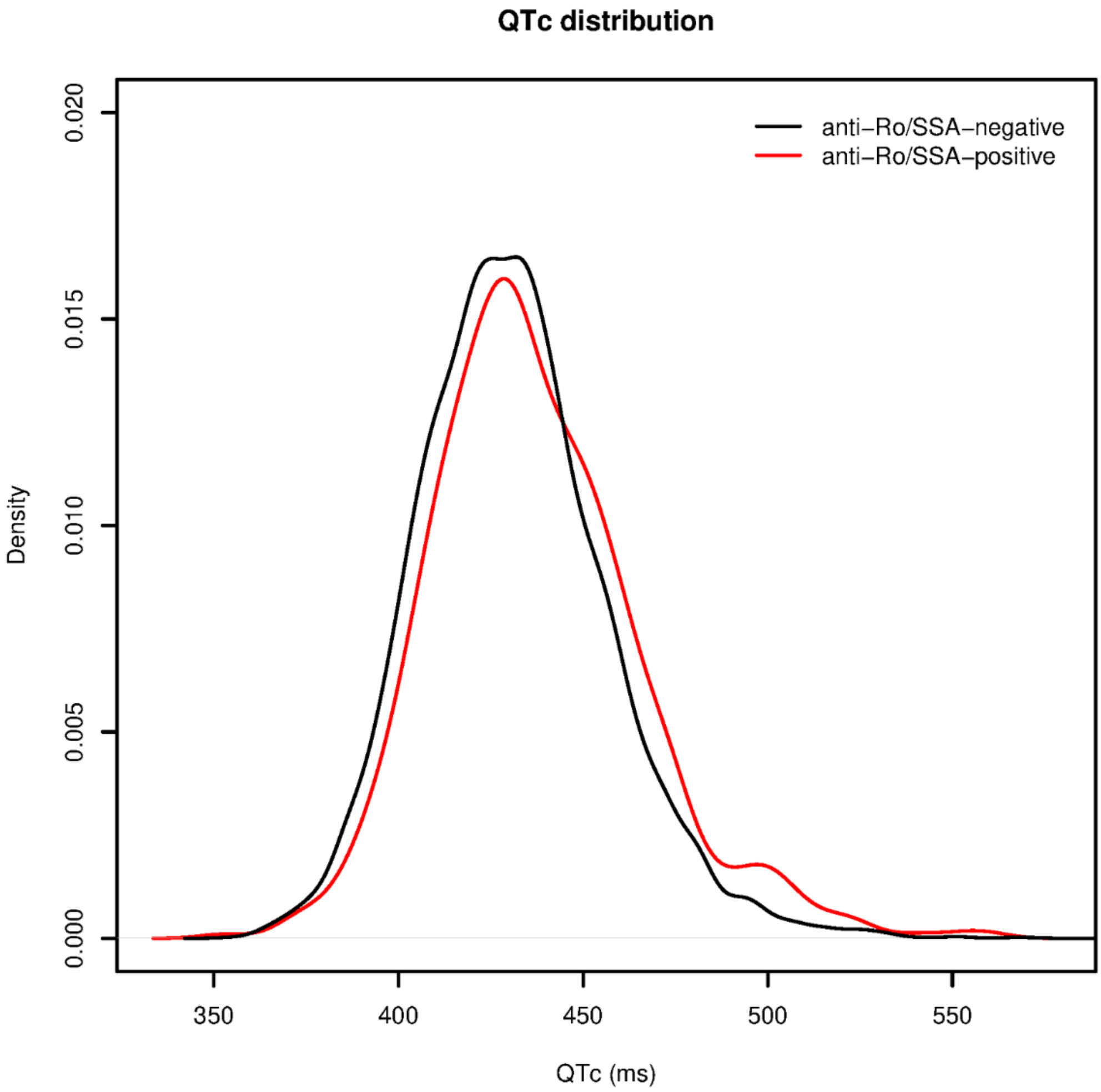


Appendix Figure 9. Empirical distribution of QTc values in a large cohort of 7,339 US veterans (*Reproduced from: Lazzerini PE, Cevenini G, Qu YS, Fabris F, El-Sherif N, Acampa M, Cartocci A, Laghi-Pasini F, Capecchi PL, Boutjdir M, Lazaro D. Risk of QTc Interval Prolongation Associated With Circulating Anti-Ro/SSA Antibodies Among US Veterans: An Observational Cohort Study. J Am Heart Assoc. 2021 Feb 16;10(4):e018735. This work is made available under the* Creative Commons CC0 *public domain dedication*)

Anti-Ro/SSA-antibodies indicates anti-Sjögren's syndrome-related antigen A-antibodies; and QTc, heart rate-corrected QT interval.

# REFERENCES

1. Ma, L.-L., Wang, Y.-Y., Yang, Z.-H., Huang, D., Weng, H., & Zeng, X.-T. (2020). Methodological quality (risk of bias) assessment tools for primary and secondary medical studies: what are they and which is better? *Military Medical Research*, *7*(1), 7. https://doi.org/10.1186/s40779-020-00238-8
2. Nomura, A., Kishimoto, M., Takahashi, O., Deshpande, G. A., Yamaguchi, K., & Okada, M. (2014). Prolongation of heart rate-corrected QT interval is a predictor of cardiac autonomic dysfunction in patients with systemic lupus erythematosus. *Rheumatology International*, *34*(5), 643–647. https://doi.org/10.1007/s00296-013-2718-z
3. Lazzerini, P. E., Acampa, M., Guideri, F., Capecchi, P. L., Campanella, V., Morozzi, G., … Laghi-Pasini, F. (2004). Prolongation of the corrected QT interval in adult patients with anti-Ro/SSA–positive connective tissue diseases. *Arthritis & Rheumatism*, *50*(4), 1248–1252. https://doi.org/10.1002/art.20130
4. Costedoat-Chalumeau, N., Amoura, Z., Hulot, J., Ghillani, P., Lechat, P., Funck-Brentano, C., & Piette, J. (2005). Corrected QT interval in anti-SSA–positive adults with connective tissue disease: Comment on the article by Lazzerini et al. *Arthritis & Rheumatism*, *52*(2), 676–677. https://doi.org/10.1002/art.20845
5. Lazzerini, P. E., Capecchi, P. L., Guideri, F., Bellisai, F., Selvi, E., Acampa, M., … Laghi-Pasini, F. (2007). Comparison of Frequency of Complex Ventricular Arrhythmias in Patients With Positive Versus Negative Anti-Ro/SSA and Connective Tissue Disease. *The American Journal of Cardiology*, *100*(6), 1029–1034. https://doi.org/10.1016/j.amjcard.2007.04.048
6. Bourré-Tessier, J., Clarke, A. E., Huynh, T., Bernatsky, S., Joseph, L., Belisle, P., & Pineau, C. A. (2011). Prolonged corrected QT interval in anti-Ro/SSA-positive adults with systemic lupus erythematosus. *Arthritis Care & Research*, *63*(7), 1031–1037. https://doi.org/10.1002/acr.20470

7. Massie, C., Hudson, M., Tatibouet, S., Steele, R., Huynh, T., Fritzler, M. J., … Pineau, C. A. (2014). Absence of an association between anti-Ro antibodies and prolonged QTc interval in systemic sclerosis: A multicenter study of 689 patients. *Seminars in Arthritis and Rheumatism*, *44*(3), 338–344. https://doi.org/10.1016/j.semarthrit.2014.07.001

8. Pisoni, C. N., Reina, S., Arakaki, D., Eimon, A., Carrizo, C., & Borda, E. (2015). Elevated IL-1β levels in anti-Ro/SSA connective tissue diseases patients with prolonged corrected QTc interval. *Clinical and Experimental Rheumatology*, *33*(5), 715–720.

9. Bourré-Tessier, J., Urowitz, M. B., Clarke, A. E., Bernatsky, S., Krantz, M. J., Huynh, T., … Pineau, C. A. (2015). Electrocardiographic Findings in Systemic Lupus Erythematosus: Data From an International Inception Cohort. *Arthritis Care & Research*, *67*(1), 128–135. https://doi.org/10.1002/acr.22370

10. Mostafavi, A. A., Taassoarian, B., Khadir, V., Abbaszadeh, S., Sanatkar, S. A., & Rafiei, M. (2020). Assessment of the Relationship Between Dose and Number of Effective Used Drugs on on QT Interval in Patients with Lupus. *Shiraz E-Medical Journal*, *21*(2). https://doi.org/10.5812/semj.83710

11. Azharudeen, M., Thabah, M. M., Satheesh, S., & Negi, V. S. (2020). Qt Interval Parameters, Anti-Ro Antibody Status, and Disease Activity in Systemic Lupus Erythematosus. *Journal of the Royal College of Physicians of Edinburgh*, *50*(4), 380–386. https://doi.org/10.4997/jrcpe.2020.406

12. Gamazo-Herrero, J., Medina-Luezas, J. A., Cusacovich, I., Martín-Asenjo, M., González-Montagut-Gómez, C., Sánchez-González, M. D., … González-Fuentes, R. (2024). Should Physicians Be Aware of Rhythm Disturbances in Adults with Systemic Autoimmune Diseases and Anti-Ro52 Antibodies? A Cross-Sectional Study. *Journal of Clinical Medicine*, *13*(12), 3510. https://doi.org/10.3390/jcm13123510

13. Villuendas, R., Martínez-Morillo, M., Juncà, G., Teniente-Serra, A., Diez, C., Heredia, S., … Olivé, A. (2021). Usefulness of cardiac screening in patients with systemic lupus

erythematosus and anti-Ro/SSA antibodies. *Lupus*, *30*(10), 1596–1602. https://doi.org/10.1177/09612033211027928

14. Lazzerini, P. E., Capecchi, P. L., Acampa, M., Morozzi, G., Bellisai, F., Bacarelli, M. R., … Laghi-Pasini, F. (2011). Anti-Ro/SSA–associated corrected QT interval prolongation in adults: The role of antibody level and specificity. *Arthritis Care & Research*, *63*(10), 1463–1470. https://doi.org/10.1002/acr.20540
15. Lazzerini, P. E., Cevenini, G., Qu, Y. S., Fabris, F., El-Sherif, N., Acampa, M., … Lazaro, D. (2021). Risk of QTc Interval Prolongation Associated With Circulating Anti-Ro/SSA Antibodies Among US Veterans: An Observational Cohort Study. *Journal of the American Heart Association*, *10*(4), e018735. https://doi.org/10.1161/JAHA.120.018735
16. Alkmim Teixeira, R., Borba, E. F., Pedrosa, A., Nishioka, S., Viana, V. S. T., Ramires, J. A., … Martinelli Filho, M. (2014). Evidence for cardiac safety and antiarrhythmic potential of chloroquine in systemic lupus erythematosus. *Europace*, *16*(6), 887–892. https://doi.org/10.1093/europace/eut290
17. Tufan, A. N., Sag, S., Oksuz, M. F., Ermurat, S., Coskun, B. N., Gullulu, M., … Dalkilic, E. (2017). Prolonged Tpeak–Tend interval in anti-Ro52 antibody-positive connective tissue diseases. *Rheumatology International*, *37*(1), 67–73. https://doi.org/10.1007/s00296-016-3488-1
18. Gordon, P. A., Rosenthal, E., Khamashta, M. A., & Hughes, G. R. (2001). Absence of conduction defects in the electrocardiograms [correction of echocardiograms] of mothers with children with congenital complete heart block. *The Journal of Rheumatology*, *28*(2), 366–369.
19. Cardoso, C. R., Sales, M. A., Papi, J. A., & Salles, G. F. (2005). QT-interval parameters are increased in systemic lupus erythematosus patients. *Lupus*, *14*(10), 846–852. https://doi.org/10.1191/0961203305lu2225oa
20. Ösken, S., Ösken, A., Polat, F., Şahin, D., Uzun, N. N., Şen, N., & Tezcan, M. E. (2025). A retrospective study of electrocardiographic alterations in primary Sjögren's syndrome: role of

anti-SSA positivity and disease duration. *Clinical Rheumatology*.

https://doi.org/10.1007/s10067-025-07477-x
